# How to prevent technostress at the digital workplace: a Delphi study

**DOI:** 10.1007/s11573-023-01159-3

**Published:** 2023-05-02

**Authors:** Michelle Berger, Ricarda Schäfer, Marco Schmidt, Christian Regal, Henner Gimpel

**Affiliations:** 1 FIM Research Center for Information Management, 86159 Augsburg, Germany; 2 Branch Business & Information Systems Engineering of the Fraunhofer FIT, Alter Postweg 101, 86159 Augsburg, Germany; 3 Department of Digital Management, University of Hohenheim, Schloss Hohenheim 1, 70599 Stuttgart, Germany; 4 University of Augsburg, Universitätsstraße 2, 86158 Augsburg, Germany

**Keywords:** Technostress, Technostress prevention, Technostress inhibitors, Technostress creators, Delphi study, M15, O33, I12

## Abstract

Technostress is a rising issue in the changing world of digital work. Technostress can cause severe adverse outcomes for individuals and organizations. Thus, organizations face the moral, legal, and economic responsibility to prevent employees’ excessive technostress. As technostress develops over time, it is crucial to prevent it throughout the process of its emergence instead of only reacting after adverse outcomes occur. Contextualizing the Theory of Preventive Stress management to technostress, we synthesize and advance existing knowledge on inhibiting technostress. We develop a set of 24 technostress prevention measures from technostress inhibitor literature, other technostress literature, and based on qualitative and quantitative contributions from a Delphi study. Based on expert feedback, we characterize each measure and, where possible, assess its relevance in addressing specific technostressors. Our paper contributes to research by transferring the Theory of Preventive Stress Management into the context of technostress and presenting specific measures to prevent technostress. This offers a complementary view to technostress inhibitors by expanding the theoretical grounding and adding a time perspective through the implementation of primary, secondary, and tertiary prevention measures. For practice, we offer a comprehensive and applicable overview of measures organizations can implement to prevent technostress.

## Introduction

Information and communication technologies (ICTs) are ubiquitous in our private and business lives. Digitalization and ICTs generally facilitate work activities and enable new ways of work (Becker et al. 2020). However, the use of ICTs may also create technostress, contributing to individuals’ overall experience of stress. Thereby, technostress describes any stress from using ICTs (Ragu-Nathan et al. 2008). It has become a severe issue with societal, economic, and personal consequences such as impaired individuals’ health or decreased work productivity (Ragu-Nathan et al. 2008; Srivastava et al. 2015). While technostress can also have positive effects—techno-eustress (Benlian 2020; Califf et al. 2020; Tarafdar et al. 2019)—we focus on the negative side, techno-distress (in this paper referred to as technostress), which is prevailing in literature (e.g., Pirkkalainen et al. 2019; Weinert et al. 2020).

Technostress is mostly described as a process, starting with *technology environmental conditions* that refer to attributes of specific ICT (e.g., push notifications) and represent a demand to the individual. The individual next appraises if the demand demonstrates a threat or a challenge, indicating a *technostressor*. This then leads to a *technostress response* (i.e., physiological, psychological, and coping responses). In the long term, the experienced technostress response can lead to serious adverse *technostress outcomes* including severe health impairments, such as burnout, depression, or exhaustion (Maier et al. 2015). Technology environmental conditions do not necessarily lead to an adverse course of the technostress process. There exists a variety of actions that can mitigate the adverse technostress outcomes throughout the technostress process. It is, therefore, crucial to not only react after negative technostress outcomes already arose.

There is a stream of literature on technostress inhibitors (Ragu-Nathan et al. 2008; Tarafdar et al. 2010, 2011, 2015; Fuglseth and Sørebø 2014; Chandra et al. 2019). Technostress inhibitors are “organizational mechanisms that have the potential to reduce the effects of technostress” (Ragu-Nathan et al. 2008: 422). Examples of technostress inhibitors include technical support provision or literacy facilitation (Ragu-Nathan et al. 2008). We build on the literature on technostress inhibitors, combine it with preventive stress management, and develop the notion of preventive technostress management.

The *Theory of Preventive Stress Management* can help to understand how throughout the technostress process, the experience of technostress can be reduced (Hargrove et al. 2011; Quick et al. 1997; Quick and Quick 1979). The theory includes a time perspective on different time windows for preventing stress. The theory recommends intervening throughout the process and divides possible measures into three categories: primary, secondary, and tertiary preventive measures (Quick et al. 1997; Quick and Quick 1979). Transferring the Theory of Preventive Stress Management into the technostress world, preventive technostress management includes *primary prevention* targeting the technostressors, thus focusing on the cause of technostress (Hargrove et al. 2011), and *secondary prevention* targeting the technostress response, thus including actions designed to improve the individual’s response (e.g., improving coping skills). *Tertiary prevention* aims to treat the adverse technostress outcomes and targets the very end of the process. By contextualizing the Theory of Preventive Stress Management to the narrower context of technostress following guidelines of Hong et al. (2014), we transfer previously generated insights in prevention theory to the technostress domain as a novel, complementary viewpoint to technostress inhibition.

Prior research in the context of preventive stress management emphasizes the role of organizations in preventing individuals’ stress and promoting employees’ and organizations’ well-being (Hargrove et al. 2011). Since many technostressors stem from ICT use at work, organizations face the moral and legal responsibility to improve employee health by preventing excessive work-related technostress. Some countries, such as Germany, have even imposed legal requirements for organizations to assess and reduce employees’ negative psychological responses (e.g., caused by technostress) at work.^1^ Not only does the prevention of technostress reduce negative consequences for employees’ health, but it saves organizations the costs of substituting employees on sick leave, among others. An analysis and comprehensive knowledge base of technostress prevention measures an organization can introduce are needed to enable organizations to reduce technostress among the employees proactively. So far, prior research has analyzed the influence of single measures (Day et al. 2012; Valta et al. 2021) or technology characteristics (Ayyagari et al. 2011; Becker et al. 2020) on selected technostressors. Given the severity of technostress’ adverse outcomes, however, research needs to provide organizations with guidance on what actionable measures exist, which they can implement to prevent technostress for their employees (Brivio et al. 2018). Hence, our research aim is to (1) identify technostress prevention measures by bringing together different strands of research, and (2) characterize them in terms of their basic approach to preventive technostress management, their applicability in practice, and their relevance in targeting technostressors.

To achieve this aim, we synthesize and advance a knowledge base of technostress prevention measures by bringing together the research strands of technostress inhibition, further technostress literature, and stress management. We use the Theory of Preventive Stress Management as a theoretical basis that we apply to technostress. We assess characteristics for the applicability of the technostress prevention measures in practice. To do so, we conduct a structured literature review on organizational measures that can prevent technostress and reframe them into actionable technostress prevention measures. We enrich the resulting list by conducting multiple focus group workshops followed by a Delphi study with industry experts, yielding 24 validated prevention measures. Based on the experts’ assessments, we produce a description for each prevention measure, a classification of several characteristics, and, in the case of primary prevention measures, an indication of the technostressors they are expected to target.

Our study advances both technostress theory and practice. We contribute to technostress literature by embedding technostress inhibitors in the larger context of preventive technostress management and by transferring the Theory of Preventive Stress Management into the specific context of technostress. Second, we provide an overview of technostress prevention measures in the research strands of technostress inhibition and related technostress literature, and develop new technostress prevention measures based on experts’ insights. Third, we enrich the understanding of all 24 measures through the characterization of their basic approach to technostress prevention (primary vs. secondary technostress prevention) and their applicability in practice. Therein, we offer a basis for understanding the different roles of prevention and provide an overview of actionable measures based on insights of related research strands. The characterization in terms of the measure’s applicability highlights the need to carefully select measures that fit the specific organizational context. Finally, our study sheds light on the dynamics underlying technostress prevention and links specific primary measures to specific technostressors, revealing that single prevention measures are no one-fits-all solutions. Overall, by uniting different perspectives on technostress prevention, we contribute towards substantial benefits for organizations, individuals, and societies by potentially reducing healthcare costs and preventing adverse personal and organizational outcomes (Maier et al. 2015; Ragu-Nathan et al. 2008; Srivastava et al. 2015).

## Theoretical background on technostress as a specific form of human stress

In their daily life, people use a large variety of ICTs, including devices such as smartphones or laptops and applications that facilitate business processes by providing tools for inter- and intra-organizational communication and collaboration (Dittes and Smolnik 2019; Zuppo 2012). ICTs shape modern work life and contribute to many positive facets of work, such as the potential to work from home or seamless collaboration across countries. For example, during the COVID-19 pandemic, ICTs allow many companies to uphold their operation despite stay-at-home and social distancing orders (Ketter et al. 2020). However, the intensive use of ICTs also risks employees’ health and performance. One of these risks is *technostress*. Technostress is a specific form of human stress that is triggered by the use of IS (Tarafdar et al. 2019). Human stress has been extensively studied and is often explained through Lazarus and Folkman’s (1984) Transactional Theory of Stress (Ayyagari et al. 2011; Ragu-Nathan et al. 2008; Tarafdar et al. 2019). The theory conceptualizes stress as a process that includes the existence of internal and external *demands* (e.g., time pressure, social conflicts), which the individual assesses in two appraisal steps. First, the individual subconsciously evaluates if the demand falls into the category of positive, irrelevant, or stressful. If categorized as stressful, the demand may threaten the individual, indicating a stressor. Next, the individual subconsciously examines if the available resources are sufficient to cope with the stressor (Lazarus and Folkman 1984). The process is therefore followed by an interdependent cycle of physiological and psychological responses (e.g., negative emotional states) and coping (i.e., behavior in response to stressors) that run in parallel, repeatedly, and cannot be separated in time (Califf et al. 2020; Lazarus and Folkman 1984; Tarafdar et al. 2019). This process, in turn, may lead to adverse outcomes such as decreased health or lower productivity.

**Fig. 1:**
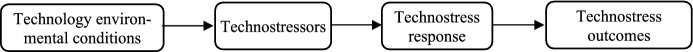
Technostress process, adapted from Fig. 2 in Tarafdar et al. (2019) and Fig. 2 in Califf et al. (2020)

Transferring the Transactional Theory of Stress into the digital world, Tarafdar et al. (2019) describe technostress as the “stress process activated due to the use of IS” (p. 8). Like stress in general, technostress can be positive (techno-eustress) or negative (techno-distress) (Tarafdar et al. 2019). In the following, we focus on the negative side of the technostress process and specifically the prevention of that negative side of technostress. The technostress process is sketched in Fig. 1 based on Califf et al. (2020) and Tarafdar et al. (2019): T*echnology environmental conditions* include potential sources of a ICT-related stressful situation. The individual exposed to such a demand from the environment appraises if the demand is harmful. If the demand is appraised as harmful it is a *technostressor*. Technostressors are “conditions or factors that can create stress because of ICT use” (Tarafdar et al. 2015: 106) and “are appraised by the individual as damaging” (Tarafdar et al. 2019: 9). When confronted with a technostressor, this leads to a multifaceted *technostress response*. The technostress response includes short-term physiological (e.g., the release of cortisol, adrenaline, and noradrenaline) and psychological (e.g., negative affect) responses that lead to coping responses (e.g., avoiding or stopping IS use).^2^ Coping response describes “actions or emotions to overcome or deal with the threat or hindrance the individual perceives from the” technostressor (Tarafdar et al. 2019: 20). Examples include seeking social or technical support (Pirkkalainen et al. 2019; Schmidt et al. 2021; Weinert et al. 2020). Coping is undoubtedly crucial to mitigate technostress but is performed entirely by the affected individual only after the technostressor has emerged. In parallel, the individuals experience a psychological or physiological response like negative emotions (e.g., anxiety, hostility) or an increase in cortisol, or a fast heart beating (Califf et al. 2020; Riedl et al. 2012). The sequence of the psychological or physiological response and the coping response cannot be clearly separated in time and can run in parallel and repetitively. Therefore, we summarize this interdependent cycle as a technostress response, consisting of psychological, physiological, and coping responses. We define *technostress response* as an interdependent, repeatedly, and potentially parallel process of negative psychological and/or physiological states caused by the technostressor (i.e., psychological and physiological response) and the application of actions or emotions to deal with the threat of the technostressor (i.e., coping response) [based on Califf et al. (2020), Tarafdar et al. (2019), Riedl et al. (2012), and Lazarus and Folkman (1984)]. In the long-term, the technostress response can lead to adverse *technostress outcomes*, especially when the technostress response is intense and long-lasting or frequently repeated. These include adverse outcomes for the individual, such as sleeping problems or emotional exhaustion – as well as adverse organizational outcomes like decreasing productivity, lower levels of job satisfaction, and less organizational commitment (e.g., Gimpel et al. 2018; Maier et al. 2015; Riedl et al. 2012; Srivastava et al. 2015).

Several technostressors have been discussed in the literature. Tarafdar et al. (2007) and Ragu-Nathan et al. (2008) were the first to develop and empirically validate scales for five technostressors: *overload*, *invasion*, *complexity*, *insecurity*, and *uncertainty*.^3^ In subsequent research, these technostressors have been applied in many studies in different contexts and are well-established today (e.g., Becker et al. 2020; Fuglseth and Sørebø 2014; Maier et al. 2019; Pirkkalainen et al. 2019). In another seminal technostress paper, Ayyagari et al. (2011) identify *unreliability*, *role ambiguity*, and *invasion of privacy* as additional technostressors. Further, Galluch et al. (2015) and Tams et al. (2018) propose ICT-enabled *interruptions* as another technostressor in work environments. While various other technostressors have been suggested in the literature (e.g., Fischer et al. 2021; Maier et al. 2012, 2015; Riedl et al. 2012), we did not consider them in our study because they are either specific to a particular technology, can be subsumed under another technostressor, or were published after the start of the Delphi study. The nine mentioned technostressors included in this research are presented in Table 1.

**Table 1:** Considered technostressors

Techno-stressor	Description
Complexity	“Situations where the complexity associated with ICTs makes users feel inadequate as far as their skills are concerned and forces them to spend time and effort in learning and understanding various aspects of ICTs” (Tarafdar et al. 2007: 315)
Insecurity	“Situations where users feel threatened about losing their jobs as a result of new ICT replacing them, or to people who have a better understanding of the ICT” (Tarafdar et al. 2007: 315)
Interruptions	Situations in which ICTs or ICT-based sources cause the user to shift their attention away from the task they are working on at that moment (Galluch et al. 2015)
Invasion	“The invasive effect of ICTs in terms of creating situations where users can potentially be reached anytime, employees feel the need to be constantly ‘connected,’ and there is a blurring between work-related and personal contexts” (Tarafdar et al. 2007: 315)
Invasion of Privacy	Situations in which users “are becoming increasingly concerned that their privacy could be invaded by computer technologies. The problem is acerbated due to the present work pressures, which create an unspoken value that appreciates individuals who are constantly available” (Ayyagari et al. 2011: 841)
Overload	“Situations where ICTs force users to work faster and longer” (Tarafdar et al. 2007: 315)
Role Ambiguity	Situations in which “there is uncertainty as to whether an individual should expend his or her resources to perform the task requirements at work or to acquire new skills. These competing demands between the job and learning new skills constrain individual abilities” (Ayyagari et al. 2011: 842)
Uncertainty	“Contexts where continuing changes and upgrades in an ICT unsettle users and create uncertainty for them, in that they have to constantly learn and educate themselves about the new ICTs.” (Tarafdar et al. 2007: 315)
Unreliability	“System malfunctions and other IT-hassles” (Fischer and Riedl 2015: 1462) that are caused by ICTs that are perceived to increase workload due to the necessity to repeat tasks (Ayyagari et al. 2011)

## Applying the theory of preventive stress management to technostress

The Theory of Preventive Stress Management, originally developed by Quick and Quick (1979), has roots in preventive medicine and public health. Nowadays, it is well established (Quick et al. 1997; Hargrove et al. 2011). The theory provides general principles of preventive stress management and tells us that there exists a variety of specific actions that can reduce stress throughout the stress process (Quick and Quick 1979). We contextualize the Theory of Preventive Stress Management to technostress (Hong et al. 2014).

Hong et al. (2014) suggest different approaches to incorporating context in theorizing. According to their typology, we engage in single-context theory contextualization with the domain of interest being technostress at work and the Theory of Preventive Stress Management as a well-established general theory. We consider concepts from both technostress theories and the Theory of Preventive Stress Management and explain their interrelations based on the underlying theory [level 2a contextualization from Hong et al. (2014)]. This leads us to the conceptual model of preventive technostress management presented in this section. In the subsequent sections, we decompose core constructs (specifically “primary technostress prevention measures” and “secondary technostress prevention measures”) into contextualized constructs (i.e., 24 specific technostress-prevention measures; level 2c contextualization from Hong et al. (2014)). In that, we follow guidelines suggested by Hong et al. (2014): We perform a thorough literature-based and expert-based evaluation of the technostress context to identify specific factors. In that, we adapt the principles of preventive stress management to the technostress context and decompose core high-level constructs of the Theory of Preventive Stress Management. As our principles of preventive technostress management and the prevention measures are manifestations of general constructs from the Theory of Preventive Stress Management, their effects can be explained based on the theoretical rationale of the general theory.

Quick and Quick (1984b) defined preventive stress management as “an organizational philosophy and set of principles that employ specific methods for promoting individual and organizational health while preventing individual and organizational distress.” It is important to note that the Theory of Preventive Stress Management takes a broader view of stress than the technostress theory. As the definition of preventive stress management shows, stress is seen as a phenomenon at the individual and organizational levels. The Theory of Preventive Stress Management considers stress an overarching rubric for how individuals and organizations react and adjust to their environments. It becomes specific for the concepts of stressor, stress response, and stress. Quick et al. (1997, p. 6) distinguish between individual stress and organizational stress, with the latter being “the degree of deviation an organization experiences from a healthy, productive level of functioning.” Stress is not bad per se – it can have positive and constructive outcomes that support performance. However, “excessive, prolonged, intense, or mismanaged stress at the workplace” result in physiological, psychological, and behavioral deviations from an individual’s healthy functioning (Quick et al. 1997, p. 18). The aggregate of this individual-level stress becomes organizational stress. This conceptualization of stress and, specifically, stress outcomes on the organizational level is uncommon for technostress research. However, it is compatible with technostress research suggesting that the organizational environment, including the technological environment, is an important determinant of technostress (e.g., Tarafdar et al. 2019) and it is in line with research on technostress inhibitors as organizational mechanisms to reduce technostress (e.g., Ragu-Nathan et al. 2008).

The Theory of Preventive Stress Management builds on five guiding principles which are applicable to technostress management (Quick et al. 1997). The five principles of (techno)stress management are:

1. Individual and organizational health are interdependent. Organizations cannot achieve goals like high productivity or flexibility without healthy individuals. This principle contributes to the need for organizations to develop preventive technostress management as part of their overall management of employee health and safety.
2. Leaders have responsibility for individual and organizational health. This leadership challenge and responsibility includes diagnosing technostress issues in the organization and selecting and implementing related technostress prevention measures. Leadership is responsible for technostress management; however, all employees are responsible for their health and their co-workers’ health.
3. Individual and organizational stress is not inevitable. The Theory of Preventive Stress Management suggests that preventive managerial actions may mitigate stress. For this, they must anticipate and influence stressors and stress processes. This paper’s practical contribution relates to the second and third guiding principles in supporting leaders in selecting adequate technostress prevention measures.
4. Each individual and organization reacts uniquely to stress. This is well in line with technostress theories and suggests that preventive technostress management needs to be tailored to specific organizations and needs to allow flexibility for individuals.
5. Organizations are ever-changing, dynamic entities. This implies that preventive technostress management cannot be a one-time effort but needs to be constantly evaluated and developed to meet the interests of the organization and the employees.

Beyond these principles, the Theory of Preventive Stress Management considers the stress process and a translated overlay which is composed of preventive interventions (Quick and Quick 1979; Quick et al. 1997; Hargrove et al. 2011). One fundamental premise of preventive medicine is that preventive measures can target each stage in the life history of a disease to slow, stop, or revert the progression of it. Applied to stress, this means preventive measures can address various points in the stress process: “Primary prevention is aimed at modifying the organizational stressors that may eventually lead to stress. Secondary prevention aims at changing individual stress responses to necessary demands. Tertiary prevention attempts to minimize the amount of individual and organizational stress that results when organizational stressors and resulting stress responses have not been adequately controlled” (Quick et al. 1997, p. 154).

**Fig. 2:**
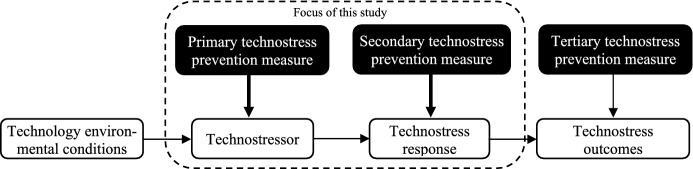
Conceptual model of preventive technostress management, adapted from Tarafdar et al. (2019), Califf et al. (2020), and Quick et al. (1997)

Building on Quick and Quick’s (1984b) definition of preventive stress management, we define *preventive technostress management* as an organizational philosophy and set of principles that employ specific measures to inhibit technostress to promote individual and organizational health. The set of five principles is described above. The measures are *technostress prevention measures*. Figure 2 positions the prevention measures along the technostress process. It is important to note that secondary and tertiary prevention are not alternatives but complements of primary technostress prevention (Quick et al. 1998). In general, excessive, prolonged, and intense technostress that will eventually lead to adverse outcomes at the individual level and, consequently, on the organizational level should be prevented as early as possible.

Technostress inhibitors and technostress prevention are related. Prevention takes measures to inhibit technostressors, technostress responses, or adverse technostress outcomes. Comparing research on technostress inhibitors to the previously presented insights on preventive stress management shows that the terms are based on the same theorization in the technostress process and refer to avoiding technostress to promote individual and organizational health. As summarized by Sarabadani (2018), technostress inhibitors are theorized to either (1) act as antecedents to technostressors, hence reducing the technostressor itself (Jena 2015; Tarafdar et al. 2011, 2015), (2) moderate the relationship between technostressor and outcomes (Ragu-Nathan et al. 2008), or (3) decrease the adverse outcomes (e.g., job satisfaction, sales performance, organizational commitment) directly (Jena 2015; Ragu-Nathan et al. 2008; Tarafdar et al. 2011). These three mechanisms of technostress inhibitors align well with the three stages of prevention (primary, secondary, and tertiary). The terminology of prevention has not been widely used in the context of technostress. Only Salo et al. (2017) used the foundation of LaMontagne et al. (2007), which is based on the Theory of Preventive Stress Management, to structure individuals’ ways of reducing technostress without introducing the term technostress prevention. Salo et al. (2017) categorized five different individual measures into stressor reduction (e.g., modification of IT features), stressor toleration (e.g., modification of personal reactions to IT stressors), and recovery from strain (e.g., online/offline venting). Building on the work of LaMontagne et al. (2007), this classification is equivalent to the classification of primary, secondary, and tertiary technostress prevention measures. However, while Salo et al. (2017) study individuals’ coping with technostress, preventive stress management theory emphasizes the duty of organizations to promote individual and organizational health and to minimize individual and organizational stress to create an organization in which their employees can thrive and produce (Hargrove et al. 2011; Quick et al. 1997). We proceed to use the term *technostress prevention measure* to build on the existing knowledge of preventive stress management. We integrate both existing knowledge on technostress inhibitors and the differentiation between primary, secondary, and tertiary preventive measures in the conceptual model of preventive technostress management (Fig. 2). The model is based on the technostress process of Tarafdar et al. (2019) and Califf et al. (2020) (Fig. 1), synthesized with the Theory of Preventive Stress Management in organizations (Quick and Quick 1979; Quick et al. 1997; Hargrove et al. 2011) (Fig. 2).

In analogy with general stress prevention (Quick et al. 1997), we characterize the three technostress prevention stages as follows: *Primary technostress prevention* is technostressor-directed. It targets reducing the frequency, duration, and/or intensity of one or multiple technostressors. The Theory of Preventive Stress Management refers to organizational stressors as it focuses on work stress. These organizational stressors result from task, role, physical, and interpersonal demands. Specifically for technostress, the organizational stressors are activated due to the use of IS (technostressors). These (techno-)stressor-directed interventions are located at the onset of the (techno-) stress process directly related to the (techno-)stressors and represent the most efficient and effective means of prevention (Quick and Quick 1979). One example of primary technostress prevention is the development of team norms or reachability rules to avoid work-related communication when not appreciated.

As primary prevention might not be effective for all individuals and in every setting, secondary stress prevention aims at improving how individuals respond to the respective stressor (Quick et al. 1997). Hence, secondary prevention is response-directed. *Secondary technostress prevention* aims at improving the individuals' technostress responses consisting of coping responses and psychological and physiological responses. An example of a secondary technostress prevention measure is providing an ICT helpdesk. At this point, the differentiation between secondary technostress prevention measures and technostress coping becomes apparent: technostress coping refers to the behavior an individual adopts when affected by a technostressor (e.g., calling the ICT helpdesk or seeking social support). Secondary technostress prevention measures are organizational-level measures that aim at improving the individual coping skills or resources and positively influence the psychological or physiological responses, for example, by offering platforms to exchange experiences on ICT use, training to improve individual coping skills, or the setup of an ICT helpdesk. Secondary technostress prevention measures do not concern the actual execution of the coping response but aim to optimize the possibility of coping.

*Tertiary technostress prevention* concerns the treatment, compensation, and rehabilitation of adverse individual or organizational technostress outcomes (Hargrove et al. 2011; Quick et al. 1997). These interventions are outcome-directed. As they intervene after the onset of adverse outcomes (e.g., burnout or depression regarding individual health outcomes), they are at the individual level mainly related to medical or psychiatric treatments. This is why we focus on primary and secondary but not tertiary technostress prevention.

Table 2 provides an overview of the key constructs of our conceptual model of work-related technostress prevention and their definitions.

**Table 2:** Summary of relevant constructs

Construct	Definition
Technostress^a^	Negative “stress process activated due to the use of IS” (Tarafdar et al. 2019) including technology environment conditions, technostressors, technostress responses, and adverse outcomes for the individual and the organization
Technology environmental conditions	Attributes or features of the information and communication technologies that surround individuals at work (Ayyagari et al. 2011; Becker et al. 2020)
Technostressors	“Conditions or factors that can create stress because of ICT use” (Tarafdar et al. 2015: 106)
Technostress response	An interdependent, repeatedly, and potentially parallel process of physiological and/or negative psychological states caused by the technostressor (i.e., physiological and psychological response) and the application of actions or emotions to deal with the threat of the technostressor (i.e., coping response) [based on Califf et al. (2020), Tarafdar et al. (2019), and Riedl et al. (2012)]
Technostress outcomes	Individual strains (i.e., physiological, psychological, and behavioral consequences) and adverse organizational outcomes caused by technostress (Ragu-Nathan et al. 2008)
Technostress inhibitors	“Organizational mechanisms that have the potential to reduce the effects of technostress” (Ragu-Nathan et al. 2008: 422)
Preventive technostress management	An organizational philosophy and set of principles that employs specific measures to inhibit technostress to promote individual and organizational health (adapted from Quick and Quick 1984b)
Primary technostress prevention	Taking measures for reducing, modifying, or managing technostressors’ frequency, duration, and/or intensity [adapted from Quick et al. (1997)]
Secondary technostress prevention	Taking measures for improving individuals’ psychological, physiological, and coping responses to technostressors [adapted from Quick et al. (1997)]
Tertiary technostress prevention	Taking measures for the treatment, compensation, and rehabilitation from adverse individual or organizational technostress outcomes [adapted from Quick et al. (1997)]

^a^Note that this is definition of technostress follows the paper’s focus on techno-distress

## Research process for identifying and characterizing primary and secondary technostress prevention measures

**Fig. 3:**
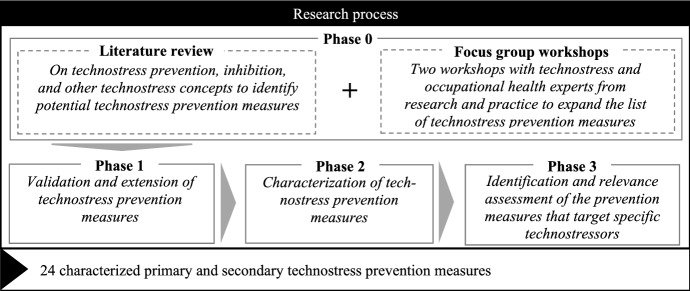
Research Process

We conducted a Delphi study with 13 experts from research and practice (Fig. 3) to achieve our research aim of (1) identifying actionable technostress prevention measures and (2) characterizing them in terms of their basic approach to technostress prevention, their applicability in practice, and in case of primary technostress prevention measures, their relevance in targeting technostressors.

To provide an appropriate starting point for the Delphi study based on theoretical and practical knowledge, we included a preliminary phase 0 to prepare a sound set of potential technostress prevention measures from literature on technostress prevention, inhibition and related technostress literature, that investigate measures to reduce the effect of technostress. Thereby, theoretical knowledge was drawn from a structured literature review (Webster and Watson 2002) on measures that may be qualified to prevent work-related technostress. To validate and expand the list of technostress prevention measures, we conducted two focus group workshops (Kitzinger 1995) with experts from research and practice before conducting phases 1–3 of the Delphi study. The central part of the Delphi study builds on these measure candidates to, again, validate, but especially to detail the technostress prevention measures. Since literature has not yet produced and documented extensive knowledge on the characteristics of technostress prevention measures, an exploratory qualitative research method is appropriate. Unlike other exploratory qualitative research methods, Delphi studies support explorative, consensus-seeking research such as problem identification, concept development, and prioritization and have become a popular research method in IS research (Okoli and Pawlowski 2004; Skinner et al. 2015). The iterative design of Delphi studies allows participants to learn from each other over a longer period of time, reflect on their opinions, and collectively develop results. The Delphi study addresses the research question by a) evaluating the list of technostress prevention measures concerning relevance, completeness, and understandability, b) characterizing the technostress prevention measures, and c) relating the primary technostress prevention measures to the nine considered technostressors presented in Sect. 2.

### Phase 0: structured literature review and focus group workshops

We conducted a structured literature review to identify known measures for reducing and eliminating technostress, which can be interpreted and converted into technostress prevention measures (Webster and Watson 2002). In theory, coping is not part of primary and secondary prevention. We nevertheless included it as a search term because some scholars provide examples of coping measures that can be used as input to develop technostress prevention measures. One reason for the lack of clear concepts could be the novelty of technostress prevention within the research stream. As suggested by vom Brocke et al. (2015) and Webster and Watson (2002), the literature review comprised three phases: (1) literature search, (2) selection of relevant literature, and (3) analysis of the results.

We defined a search string, databases, and inclusion and exclusion criteria (vom Brocke et al. 2015). As the literature on technostress prevention is highly dispersed and often described under different terminology, we chose a broad search string that considers adjacent concepts and literature from various disciplines (information systems, organizational science, health, and psychology). Therefore, we searched for topics, abstracts, titles, and keywords that matched the search string ((“technostress” OR “techno stress” OR “digital stress”) AND (“prevent*” OR “reduc*” OR “mitigat*” OR “overcome” OR “cop*” OR “inhibit*”)) in the three databases Web of Science, PubMed, and AISeL as well as in the Journal of Business Economics. In total, we found 204 articles. After filtering for English, peer-reviewed, full research articles (n = 192), removing duplicates (n = 167), and adding previously known articles according to Larsen et al. (2019) our set consisted of 171 articles. Based on our selection criteria, we conducted a three-step selection process, including title, abstract, and full-text review (Levy and Ellis 2006; Okoli and Schabram 2010). The selection criteria included: (1) the article is within the domain of technostress, and (2) the article includes at least one recommendation for preventing technostress (at work/in organizations) following our definition of technostress prevention measures. After the title screening, 102 articles remained. The abstract screening excluded an additional 42 articles, resulting in 60 articles that went into detailed analysis. For details, please see Fig. 5 in the appendix. Finally, the full-text screening resulted in a list of 40 relevant articles out of which seven articles belong to the specific domain of technostress inhibition, while the remaining 33 articles result from the additional terms in the search string (Table 5 and Table 6 in the appendix).

Independently from the literature review, we conducted two focus group workshops with technostress and occupational health experts from research and practice (Table 8 in the appendix) to potentially expand the list of technostress prevention measures. The workshops aimed to identify recent or modern concepts for dealing with technostress that are being applied in practice but are not (yet) embedded in literature. Therefore, in the first focus group workshop, eight experts from practice and four from research developed a set of technostress prevention measures guided by a moderator and a minute taker (Conklin and Hayhoe 2010). The participants’ areas of expertise include information systems, psychology, and occupational health and safety. The workshop’s procedure was inspired by Then et al. (2015). The participants collected and discussed possible technostress prevention measures that organizations can take by making targeted changes to technologies, organizations, or individuals. A second moderated workshop with five experts (four from practice, one from research) expanded on the first workshop’s results. The participants were introduced to the technostressors to develop adequate technostress prevention measures. To guarantee the privacy and foster an open atmosphere, we refrained from recording and transcribing the workshops. Instead, we created a photo protocol by taking photographs of the final whiteboards and collected the focus groups’ notes as field notes (Miles and Huberman 1994)—a valid qualitative data source in workshops (Ørngreen and Levinsen 2017). Based on this information, the moderators of the two workshops summarized the results and prepared them for the participants. The participants then had the opportunity to add to or adjust the summaries until all participants were satisfied.

With the two workshops, we collected 94 technostress prevention measure candidates on different levels of detail. From the 40 relevant literature articles, we extracted 34 distinct recommendations for preventing technostress (Tables 5 and 6 in the appendix). Two researchers jointly categorized the measure candidates, grouped them on the same level of detail, and fitted them to our definition of technostress prevention. This procedure yielded an initial list of 24 technostress prevention measures that went into the Delphi study. We used the detailed technostress prevention measure candidates to describe and exemplify the aggregated technostress prevention measures. We formulated the underlying inhibiting effect and a measure’s description based on both input sources during this process.

### Phases 1, 2, and 3: The Delphi study

Our study is a slightly modified ranking-type Delphi study (Schmidt 1997), a common Delphi study approach (Paré et al. 2013). The ranking-type Delphi study proposed by Schmidt (1997) consists of three phases: (1) brainstorming to discover the issues, (2) narrowing down to determine the most important issues, and (3) ranking the issues. Similar to other published Delphi studies (Paré et al. 2013), we merged the first and second phases because the literature review and the focus group workshops in phase 0 already structured the topic. Instead, we added a phase for characterizing each technostress prevention measure. Each phase consisted of two rounds to validate the previous round’s result, which is appropriate for producing credible results (Skinner et al. 2015). Consequently, our Delphi study consisted of three phases:

1. *Validation and extension of technostress prevention measures*
2. *Characterization of technostress prevention measures*
3. *Identification and relevance assessment of primary technostress prevention measures that target specific technostressors*

To ensure that the participants of our Delphi study are knowledgeable experts in technostress and are aware of the issue in the larger world around them (Delbecq et al. 1975; Keeney et al. 2006; Skinner et al. 2015), we applied the following selection criteria (Okoli and Pawlowski 2004): experts should (1) be responsible for, for example, occupational safety/medicine, psychological risk assessment/operational health management, or human resources, (2) have experience in the field of technostress, stress management, or occupational health and safety, (3) have at least three years of work experience, and (4) be frequent users of ICTs themselves. These requirements ensured that all experts were familiar with technostress and possible technostress prevention measures. We ensured that all experts professionally deal with stress management programs (all work on general occupational stress management; some focus on technostress management). Not every expert had experience with all measures presented but at least with multiple technostress prevention measures. Thus, they can assess the measures, evaluate them, and estimate the implementation. Experts had the choice not to provide assessments on individual measures when they felt they had insufficient information or experience with the measure. But this option was never used in the Delphi study. We have no indication for systematic differences in the assessment of measures by the experts depending on the expert’s first-hand experience with a measure as compared to only abstract knowledge about the measure. Such differences could have occurred in phases 2 to 3. In the progression of the Delphi study, the experts’ assessments tended to converge, given even less basis to differentiate by experience. Hence, we report aggregate results from the entire panel.

To ensure broad topic coverage, we recruited experts from different industries and company sizes via our industrial network and several occupational health and safety events on the topic of technostress. We reached out to a total of 50 possible experts. Of those, 15 experts fulfilled the selection criteria and agreed to participate in our Delphi study. None of them participated in the focus group workshops in Phase 0. One participant missed the first round, and another dropped out after the first round. Thus, 13 experts completed each round, an appropriate panel size (Linstone and Turoff 2002; Paré et al. 2013). Table 8 in the appendix provides additional information on the panel.

The Delphi study took place via the online survey tool LimeSurvey and lasted five months (August to December 2020). In each round, we invited all experts to participate via e-mail and provided detailed instructions. To provide the experts with sufficient guidance, we e-mailed them their answers and suggestions from the previous round, an overview of the changes made based on all experts’ suggestions, and descriptive information on quantitative evaluations after each round. Thereby, the experts could reflect on their opinion based on the aggregated results from all experts. Also, experts could provide free text feedback in addition to quantitative assessments. Through the free text feedback, we also provide the opportunity to state that one has had too little experience with the measure for assessing it. Following the suggestion of Strasser (2016), Okoli and Pawlowski (2004), and Skinner et al. (2015), participants were not known to each other by name, and we did not show the individual results of other panelists to ensure anonymity and avoid bias.

*Phase 1* introduced the experts to the 24 technostress prevention measure candidates and suggested a description of each measure and their inhibiting effects. We asked the experts to rate each technostress prevention measure’s relevance for preventing technostress on a scale ranging from 0 (”not relevant”) to 6 (“highly relevant”). Literature on the Delphi method suggests to identify the key issues (Paré et al. 2013) or most important issues (Schmidt 1997) in the respective context. In our study, this means identifying the most relevant prevention measures. Relevance in general refers to (1) a relation between two entities in a context, (2) to an intention, and (3) to an assessment of the effectiveness of the relation regarding the intention (Cosijn and Ingwersen 2000; Saracevic 2007). In our study, the context is technostress at work, and the relation is between technostress prevention measures and parts of the technostress process. Specifically, primary prevention measures relate to technostressors, while secondary prevention measures relate to technostress responses (also see Fig. 2 for the underlying model). The intention is to prevent technostress. Effectiveness relates to the ability of a measure to reduce technostress, i.e., mitigate technostressors (primary prevention) or mitigate negative stress responses (secondary prevention). Hence, we define the relevance of a technostress prevention measure as the assessment of the measure’s effectiveness in preventing the negative effects of technostress. The experts’ judgment on relevance thus inherently relates to the expected effectiveness of a prevention measure. Directly assessing effectiveness is not possible in a Delphi study. Assessing expected or perceived effectiveness would in principle be possible in a Delphi study. However, using the term effectiveness might be perceived to suggest a level of precision of measurement that our Delphi study could not deliver. Hence, we asked the experts to assess the broader concept of relevance.

**Fig. 4:**
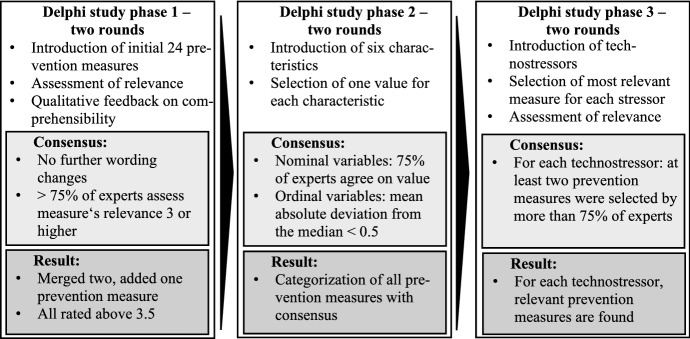
Phases 1, 2, and 3 of the Delphi study

**Fig. 5:**
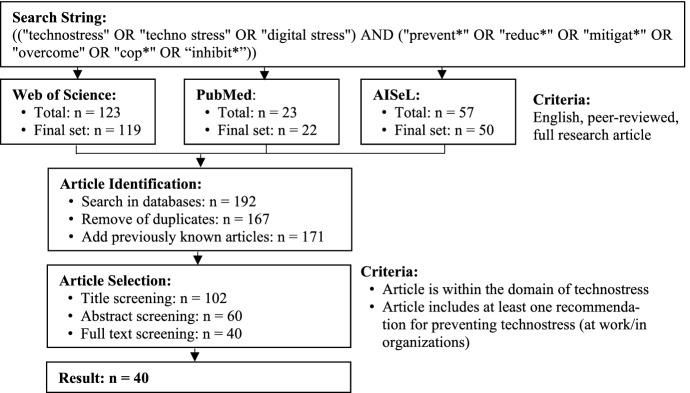
Literature review process

We queried qualitative feedback regarding each measure’s evaluation and overall comprehensibility by providing optional text fields. Additionally, the experts were asked to review the entire list, emphasizing its completeness regarding the most relevant technostress prevention measures and adding further measures if necessary. For evaluating this phase, we examined the written qualitative feedback and the relevance assessment. We defined consensus on the technostress prevention measures’ relevance as a state in which (1) the experts mention no further wording changes or new technostress prevention measures and (2) more than 75% of the experts assess each measure’s relevance with a score of 3 (middle option) or higher. To avoid bias by individual experts, recommendations for description changes or additions of technostress prevention measures were only implemented if at least two experts made suggestions in that direction. The experts did not yet reach a consensus in the first round. Based on their feedback, two measures of the initial set were merged, and one new measure was added. In the second round, we presented the aggregated results of the first round to the experts. This round resulted in a consensus, yielding the final set of technostress prevention measures. To take a first step toward the technostress prevention measures’ categorization, three authors separately grouped the technostress prevention measures into primary and secondary preventions based on the measures’ descriptions. For each measure with different categorizations of the three researchers, they discussed them together until they agreed. Figure 4 summaries key aspects of this first phase and the following phases.

*Phase* 2 introduced the experts to different characteristics of technostress prevention measures through short explanatory descriptions and figures. The characteristics were developed by the research team with the help of practitioners from organizational health and safety and represent a valuable step to better understand technostress prevention. Each expert could indicate one manifestation for each characteristic. The six characteristics are:

1. *Entity of change* describes whether the technostress prevention measure alters an organization’s I) technology, II) organizational structures and procedures, or III) the individuals’ skills and abilities [based on Murphy and Sauter (2004)]. Technology, organizational structures, and organizational procedures relate to the technology environment conditions. Individual skills and abilities relate to the individual perceiving and assessing demands from the environment. Each of the three levels refers to the entity affected by the measure, which is always implemented by the organization to prevent technostress (in line with principle 3 of (techno)stress prevention; see Sect. 3).
2. *Target group* describes whether the technostress prevention measure targets I) all employees, II) management only, or III) other specific groups. This characteristic again relates to Principle 4 of (techno)stress prevention management as the first step to account for the heterogeneity of individuals. Specifically considering management relates to principle 2 which places a specific responsibility on the leadership team that serves as a role model and determines essential aspects of the employees’ work environment.
3. *Organization size* determines if the technostress prevention measure is suitable for organizations with at least I) one or more, II) ten or more, III) 50 or more, IV) 250 or more, V) 500 or more, or VI) 2,500 or more employees. Principle 4 of (techno)stress prevention management suggests that not all organizations are alike. Including the organization size is one broad category in the direction of accounting for the heterogeneity of organizations.
4. *Duration of implementation and time to operational use* distinguishes between technostress prevention measures that take (I) less than 1 year, I(I) 1–3 years, or (III) more than three years to become operational. While the principles of (techno)stress management suggest that stress is not inevitable (principle (3) and that leaders should select and implement technostress prevention measures, this process cannot happen instantaneously. Characterizing the duration between the selection of a prevention measure to be implemented until this measure is operational captures one element of time lack in realizing the effects of technostress prevention management.
5. *Time until the effect of the technostress prevention measure is realized in the operational business* can take (I) less than half a year, (II) 0.5–1 year, or (III) more than one year. Individual and organizational health are interdependent (principle 1), and both individuals and organizations react (principle 4). However, this reaction is not instantaneous upon the use of prevention measures but may take time. Characterizing the time from the operational use of a prevention measure until the effects of the measure are realized on the induvial and organizational levels is the second element of time lack in realizing the effects of technostress prevention management.
6. *Duration of the technostress prevention measure’s effect* may be (I) less than 1 year, (II) 1–3 years, or (III) more than three years. A technostress prevention measure implemented may have long-term effects but the effect may also fade out. One reason is that organizations are ever-changing, dynamic entities (principle 5). Hence, characterizing the duration of the effect aims at informing technostress prevention management regarding the likely need to repeatedly reassess the state of technostress and its prevention in an organization.

In both rounds of Phase 2, we evaluated the results based on the characteristics’ mode (for nominal characteristics 1 and 2) and the characteristics’ median (for ordinal characteristics 3, 4, 5, and 6). For characteristics with a nominal scale, the consensus is reached if 75% of the experts agree on the same characteristic (i.e., the mode). For characteristics with an ordinal scale, we defined consensus as an average absolute deviation from the median below 0.5. To further evaluate the consensus between the experts, we calculated Fleiss’ Kappa for all six characteristics. The received values range from 0.01 to 0.43 in round 1 of Phase 2. Here, experts had not yet consensus. In round 2 of Phase 2, Fleiss’ Kappa increased to 0.79–1.00. Except for organization size (0.79, substantial agreement), all Fleiss’ Kappa values indicate an almost perfect agreement (Landis and Koch 1977). Again, consensus on the characterization of the technostress prevention measures was reached after two rounds.

In *Phase 3,* the experts were given a definition and an illustrative example of each technostressor as presented by Gimpel et al. (2020). The experts then selected the prevention measures expected to be most relevant for mitigating each technostressor. In the following, we discuss the relevance of prevention measures for specific technostressors. As the results are based on expert assessments in a small sample, we do not indicate absolute and quantifiable relationships but use relevance as a measure for giving a trend statement on the expected relationship. The trend statements serve as the foundation for potential statistical analyses in the future.

For primary technostress prevention measures, this means that the measure is expected to reduce the corresponding technostressor directly. For secondary prevention measures, we do not present this relationship because one technostressor can trigger several technostress responses and one technostress response can be triggered several technostressors. Therefore, the association of the relevance relationship between secondary prevention measures and technostressor is not meaningful. Based on the results, we assess both the set of primary technostress prevention measures relevant for each technostressor as well as the set of technostressors to which a single primary technostress prevention measure is expected to be relevant. We asked the experts to assess the relevance of the selected technostress prevention measures for the specific technostressor on a scale ranging from 0 (“not relevant”) to 6 (“highly relevant”). To evaluate the connections between technostressors and technostress prevention measures, we counted how often each technostress prevention measure was selected for each of the nine considered technostressors. We declared consensus when five or more technostress prevention measures were selected by more than 75% of the experts (that is, at least ten experts). In the second round, all technostressors met this criterion. Accordingly, the Fleiss’ Kappa values indicating the degree of agreement between the experts for each of the nine considered technostressors increased from 0.09 to 0.33 in round 1 to 0.72 (substantial agreement) to 0.94 (almost perfect agreement) in round 2 of Phase 3.

Lastly, based on our overall results, we present propositions that contain insights gained through applying the Theory of Preventive Stress Management to technostress and knowledge developed throughout our structured literature review, focus group workshops, and the Delphi study.

## Results on primary and secondary technostress prevention measures

### Overview on technostress prevention measures

Our research process resulted in a list of 24 technostress prevention measures, out of which 17 measures can be categorized as primary technostress prevention and seven as secondary technostress prevention measures (Table 3). Primary technostress prevention measures include, for example, the implementation of reachability management. By establishing reachability management, such as defining when employees are available for work-related communication, technostressors can be reduced. In contrast, secondary technostress prevention measures include the provision of ICT support. Assisting employees with fast and competent support for technical issues using ICTs allows them to better response to technostressors. The individual technostress response consists of coping responses and psychological and physiological responses (see Sect. 3).

**Table 3:** Technostress prevention measures that are sorted by their basic approach to technostress prevention—the column ‘Inhibition?’ indicates if the measure has been mentioned in any technostress inhibition article, ‘#’ indicates how many times the measure (e.g., input that helped to formulate the measure) was mentioned in the literature

No	Technostress Prevention Measure	Inhibition?	#	Inhibiting Effect	Description
**Primary technostress prevention measures**					
*Enabling changes to technology*					
1	Focus the ICT landscape	No	– (new)	Establishing an ICT landscape that meets job-specific requirements can reduce the frequency, duration, and/or intensity of technostressors	The company reduces the selection of available technologies to a reasonable level and avoids redundancies of systems and information. Employees are involved in the selection process and can use the appropriate media for a given situation (e.g., a chat for informal agreements)
2	Adapt a stress-sensitive digital workplace design	No	2	Adapting a digital workplace to the needs of employees can reduce the frequency, duration, and/or intensity of technostressors	The design of workplaces in the organization regards ergonomic aspects and technostressors, for example, by integrating rest areas and (digitally enabled) creative or group rooms. As a result, employees can use an environment suitable for their individual digital work situations
3	Apply human-centered release management	Yes	7	Establishing good planning and consideration of employees’ needs for technology-related changes in the form of updates can reduce the frequency, duration, and/or intensity of technostressors	For good release management, the company bundles changes to ICTs and works towards an effective ICT infrastructure. The change of ICTs is oriented towards the employees' needs, which are collected through formats such as the helpdesk or the mentor. Likewise, “inexperienced” employees are involved in the survey of needs. The effectiveness of the ICT infrastructure is regularly reviewed and provided in sufficient capacity
4	Apply human-centered ICT design	Yes	5	Tailoring ICTs to the needs of employees can reduce the frequency, duration, and/or intensity of technostressors	ICTs are designed regarding the reduction of technostressors and are improved through continuous user involvement. Technologies that are intuitive to use and ergonomic are preferred. For example, inefficient ICT interfaces are reduced, and work-relevant information is made accessible barrier-free and straightforward. Employees have an active involvement in changes at an early stage
5	Use gamification	No	– (new)	Using playful and rewarding elements in ICTs can reduce the frequency, duration, and/or intensity of technostressors	ICTs are expanded in a reasonable scope to include playful elements that motivate employees, for example, by collecting points for the use of ICTs. The use of gamification should be implemented voluntarily and professionally to avoid performance monitoring
*Enabling changes to organizational structures and routines*					
6	Foster a cooperative culture	Yes	8	Fostering a cooperative (rather than competitive) culture with digitalization as a common goal can reduce the frequency, duration, and/or intensity of technostressors	The company defines guiding principles and develops and establishes a digital-compatible and cooperative culture. Employees’ active involvement and role model by managers help implement the culture long-term and sustainably
7	Develop a mission statement for digital collaboration	No	5	Fostering open communication and high transparency regarding the requirements and expectations in dealing with ICTs can reduce the frequency, duration, and/or intensity of technostressors	An interdisciplinary team develops a company-wide mission statement for digital collaboration in a participatory way. The way of communication through and with ICTs will be clearly defined company-wide and followed up in the long term
8	Introduce an employee data security concept	No	1	Providing transparency over how work-related data collected by technology is processed and used, particularly in performance monitoring, can reduce the frequency, duration, and/or intensity of technostressors	The company introduces a data security concept regarding the accessibility, use, and processing of information and data regarding employees’ behavior. The concept is regularly communicated transparently to the workforce. The data security concept is revised and updated regularly to ensure that it is up to date
9	Agree on binding ICT usage guidelines	No	– (new)	Providing transparency regarding which technologies are used for which purpose helps employees avoid technostress. Joint guidelines from employee representatives and management increase acceptance among the entire staff, which can reduce the frequency, duration, and/or intensity of technostressors	Binding ICT usage guidelines are concluded within the company that clearly defines the goals, purposes, and framework conditions for using ICTs. The guidelines are revised and updated regularly to ensure that they are up to date
10	Consciously manage ICT-related change	Yes	1	Providing good and structured support with technology-related change processes can reduce the frequency, duration, and/or intensity of technostressors	The company actively adapts ICTs to changing internal requirements following good change management. Employees are informed early and comprehensively about the reasons and consequences of the change and can efficiently continue using the changing technologies through training
11	Develop team norms for the use of ICTs	Yes	7	Explicitly communicating rules for handling ICTs for team-internal tasks (e.g., preferred communication channels or file storage) can reduce the frequency, duration, and/or intensity of technostressors	The team develops rules and guidelines for handling ICTs derived from the company-wide guidelines during team-internal workshops. These rules will be explicitly communicated, documented, and passed on to new employees and continually refined in the long term in an interactive process, allowing quick adaptions. This includes, for example, the definition of rules on which communication tool should be used for which purpose
12	Establish reachability management	No	2	Creating a common understanding of when, why, and how employees are available for work-related communication can reduce the frequency, duration, and/or intensity of technostressors	The company defines clear reachability rules, which specify under what conditions and when ICTs are used. These rules are agreed upon together in collective agreements (where there is employee representation). Reachability rules are considered when selecting suitable technologies, for example, to enable the selection of unavailable times
*Enabling changes to individuals*					
13	Train managers to successfully lead in the digital working world	Yes	12	Having digitalization-friendly, inspiring, and employee-oriented leadership can build a trusting and supportive relationship between managers and team members, thus can reduce the frequency, duration, and/or intensity of technostressors	The company prepares managers for digitalization challenges in workshops by jointly developing key aspects of digital employee management. For example, this includes supporting employees by making time and other resources available to facilitate effective self- and time management
14	Train managers for leading distributed team members	No	1	Having good skills in coordinating distributed teams among managers can reduce the frequency, duration, and/or intensity of technostressors	The company offers workshops for managers to develop important aspects of leading distributed teams and train in the effective coordination and organization of tasks with ICTs
15	Provide role models with technological changes	Yes	6	Showing role models in the healthy use of ICTs and effective support of employees in the event of changes can reduce the frequency, duration, and/or intensity of technostressors	The company offers workshops for managers to train the healthy use of ICTs and an appropriate way of dealing with change processes. In this way, managers exemplify the use of the newly introduced technologies and serve as role models (e.g., regarding reachability expectations), and support employees in dealing with change
16	Train mentors for digital topics	Yes	5	Providing a personal mentor, who assists employees with technical questions, can reduce the frequency, duration, and/or intensity of technostressors	Employees can request a mentor as a trusted contact person for questions on digital and technical issues. The mentor regularly provides tips and tricks for using ICTs. The inhibition threshold to ask questions is lowered
17	Train effective self-management and time management	No	10	Practicing good self and time management skills can reduce the frequency, duration, and/or intensity of technostressors	The company offers voluntary training courses that introduce important aspects of self-management and time management regarding their digital working style. This training includes integrating short breaks into the daily work routine to prevent fatigue and a drop in performance. It enables employees to use ICTs independently, efficiently, and in a beneficial way to their health
**Secondary technostress prevention measures**					
*Enabling changes to technology*					
18	Provide supportive ICTs	No	7	Providing ICTs that support employees in their technostress response, especially in adopting new work routines as coping measures	Employees can access ICTs with functions that can support them in dealing with technostressors (i.e., changing their technostress response). These include, for example, reminders of breaks, exercise, or important tasks
*Enabling changes to organizational structures and routines*					
19	Provide ICT support	Yes	12	Providing competent, fast, and empathetic support for technical questions and problems that allows employees to change their technostress response	The company sets up a helpdesk to provide employees with fast and competent support for technical questions or problems using ICTs. The helpdesk is trained in the avoidance of technostress and, to purely first-level support, can also respond to requests from overburdened ICT users so that they feel they are taken seriously. All employees know that they can get help from the helpdesk without reproach
*Enabling changes to individuals*					
20	Train monotasking	No	3	Offering training to focus on only one task at a time helps employees to change their technostress response	The company offers voluntary training courses that inform employees about the advantages of monotasking – that is, focusing on only one task at a time –, teach approaches to efficient work design, and accompany employees’ testing of these approaches in everyday work
21	Train technostress coping competencies	No	– (new)	Providing technostress coping training help employees to change their technostress response	The company offers voluntary technostress coping training to their employees that enables them to reduce or eliminate strain caused by technostress. These measures include, for example, the conscious use of ICTs or the avoidance of stressful ICT characteristics
22	Offer platforms to exchange experience on ICT use	Yes	5	Offering sharing formats that enable employees to exchange information on how to use ICTs help employees to change their technostress response by strengthening community sense	The company establishes forums (preferably in person or as a hybrid format), through which employees can exchange experiences and best practices in dealing with ICTs. Experts moderate these platforms to facilitate joint learning
23	Provide ICT training	Yes	14	Providing training in in-depth technical skills and strengthening media competence helps employees to change their technostress response	The company offers voluntary training courses that teach employees advanced skills in the use of ICTs. During several pieces of training, employees learn how to use ICTs and practice the transfer in their daily work. These courses are offered especially when new technologies are introduced. Company-wide regulations ensure that every employee is entitled to this training
24	Foster sensitization and self-reflection regarding technostress	Yes	11	Supporting awareness of the causes, effects, and outcomes of technostress and one’s working methods helps employees to change their technostress response	The company offers voluntary training courses that sensitize employees to the dangers of technostress and teach them a toolbox of targeted strategies that help avoid technostress. Exemplary tools include introspection and self-reflection strategies to identify pitfalls of digital work and adapt their way of working

‘–’ indicates that the measure is self-developed

Table 3 presents the complete list of the 24 technostress prevention measures. The column “Inhibition?” indicates whether we extracted this measure from technostress inhibitor literature. The seven technostress inhibitor articles (see Table 5 in the appendix for details) each suggested two to seven technostress prevention measures with some overlap among the articles. Overall, twelve prevention measures mainly result from technostress inhibition literature. An additional eight measures result from the remaining articles we reviewed during Phase 0. Finally, four measures are self-developed during our research process bases on experts’ feedback. Examples for measures that result, among others, from technostress inhibition article by Tarafdar et al. (2015) is measure 19 *provide ICT support* or measure 23 *provide ICT training*. An example for measures that resulted from remaining literature besides technostress inhibition literature, is measure 2, *adapt a stress-sensitive digital workplace design*. Measure 2 results from prevention measure candidates from Arnetz (1996) and Lee et al. (2014), who do not refer to technostress inhibition but still provide valuable insights for the development of technostress prevention measures. The column “*#*” indicates how many times the technostress prevention measure was mentioned in the literature; “–” indicates that the technostress prevention measure was self-developed. The four self-developed measures are measure 1, *focus the ICT landscape*, measure 5 *use gamification*, measure 10 *agree on binding ICT usage guidelines*, and measure 21 *train technostress coping competencies.* While measures 1, 5, and 10 were developed during the workshops in Phase 0, measure 21 was added based on experts’ suggestions in Phase 1 of the Delphi study.

Measure 1 refers to the situation of having several redundant systems for the same task (e.g., video conferencing) and information distributed in the organization. The measure aims at reducing the number of systems to a reasonable level and especially involves employees in selecting appropriate systems and ICTs for the given task (e.g., to also offer room for practices like “bring your own device” if this simplifies the process and collaboration). Measure 5 *use gamification* targets the ICT design by including game elements, for example, levels, points, rewards, or badges. ICTs might also react with humor in certain situations. In this way, users are introduced to ICTs through playful behavior. It is important to note that through the Delphi study, we found that participation in such games should be voluntary to avoid (perceived) performance monitoring. Measure 10 *agree on binding ICT usage guidelines* goes in a similar direction as measure 1 but emphasizes the importance of transparency on which ICT is used for which purpose and under what conditions. Measure 21 *train technostress coping competencies* is also highly relevant in the Theory of Preventive Stress Management and separates the constructs of coping from preventive technostress measures (Quick et al. 1997). Sufficient coping skills are highly important to effectively deal with technostress (Salo et al. 2020; Schmidt et al. 2021; Weinert et al. 2020). The preventive measure trains employees’ individual technostress coping behavior being part of their technostress response. Coping includes, for example, positive reappraisal, changing the perception of an IT event, or seeking help from colleagues (Beaudry and Pinsonneault 2005, 2010).

In comparison to the technostress prevention measures synthesized from literature during phase 0, we merged the two technostress prevention measure candidates *foster sensitization regarding technostress* and *foster self-reflection regarding technostress* into *foster sensitization and self-reflection regarding technostress* based on experts’ input during the Delphi study (measure 24, Table 3), and also added a new measure 21, as mentioned above. Besides these apparent changes to the list of technostress prevention measures, the experts’ qualitative feedback in the Delphi study’s first phase suggested changes concerning the configurations of single technostress prevention measures. One expert, for example, pointed to the importance that, “especially for inexperienced employees with an IT problem, the IT helpdesk [measure 19, *provide ICT support*; note from the authors] staff should convey a feeling of trust and respect”. Many of these suggestions are reflected in the descriptions of the measures. In addition, multiple experts stressed that organizational offers suggested by measures 17 (*train effective self-management and time management*) and 24 (*foster sensitization and self-reflection regarding technostress*) should not be mandatory, as that might even increase employees’ stress.

In addition, various experts emphasized the relevance of a diverse portfolio of measures. Although measures targeting individual-level change are important, these measures bear the risk of outsourcing the responsibility for technostress prevention from the organization to the individual. Therefore, the complementary implementation of organizational and technical changes is essential. The 24 technostress prevention measures give a comprehensive overview of available measures in organizational settings and indicate the diversity of technostress prevention opportunities. The list of technostress prevention measures is structured along with the differentiation into primary and secondary prevention and the distinction between measures enabling changes to technologies, organizations, or individuals. The overview of references referring to the measures is presented in the appendix in Tables 5 and 6.

### Characterization of technostress prevention measures

To better structure and compare the different types of technostress prevention measures, experts characterized them on a set of characteristics during the Delphi study. Each technostress prevention measure’s complete characterization is presented in Table 9 in the appendix. The first characteristic (*entity of change*) relates to the entity affected by the technostress prevention measure and comprises the technological, organizational, and individual levels (Murphy and Sauter 2004). This differentiation stems from Murphy and Sauter's (2004) effort to structure interventions that avoid negative influences on worker health and safety. Measures at the technological level concern the implementation and use of well-designed ICTs that serve their purpose. At the organizational level, measures focus on changing organizational structures, processes, and guidelines (e.g., code of conduct and operating instructions). Lastly, changes on the individual level comprise technostress prevention measures that create a change in the individual, for example, their behavior. The manifestations of this characteristic are distributed relatively evenly across the 24 measures. The subheadings in Table 3 structure the classification (e.g., measures underneath the subheading “Enabling Changes to Individuals” refer to measures that affect the individual level). Six of the 24 measures address the technological level (e.g., measure 3, *apply human-centered release management*), eight address the organizational level (e.g., measure 12, *establish reachability management*), and ten address the individual level of prevention (e.g., measure 17, *train effective self-management and time management*). Technostress prevention measures on all three levels (technological, organizational, individual) can act as primary or secondary prevention (Murphy and Sauter 2004; Pirkkalainen et al. 2019; Salo et al. 2017; Weinert et al. 2020). However, the characterization shows very different frequencies of the three levels when comparing the two types of prevention. For primary prevention, the technological level, the organizational level, and the individual level are addressed by an about even number of primary prevention measures (5, 7, and 5 respectively). In contrast, secondary technostress prevention measures primarily address the individual (1, 1, and 5 measures for the technological, organizational, and individual levels, respectively).

Primary technostress prevention measures on the technological level alter the technological environment in such a way that employees experience fewer technology environmental conditions perceived as demanding. Complementary, secondary technostress prevention measures on the technological level refer to measures that aim at providing employees with technological resources to improve their response to technostress (e.g., helping employees by reminding them to take breaks). Similar accounts for primary and secondary prevention measures that target the organizational environment. While individual-level secondary technostress prevention measures mainly address the techno-stressed individual’s internal factors (e.g., knowledge, skills, experience), individual-level primary technostress prevention measures primarily target a change of other individuals’ behaviors, thus, shaping the social environment.

*Target group* and *organization size* concern the applicability of the measure for specific people and organizations. On the characteristic of the target group, we observe no fundamental differences between primary and secondary technostress prevention. In terms of the appropriate organization size, nine measures are assessed to be suitable for organizations with as few as ten employees, 13 measures for at least 50 employees, and two measures require at least 250 employees to be conducted effectively. While most measures in our study (21) are seen as suitable to all employees, three are relevant to management only. Interestingly, most primary technostress prevention measures (13/17, ~ 76%) are deemed applicable only for organizations with at least 50 employees. With secondary prevention, five of seven measures (~ 71%) are already applicable with as few as ten employees. This indicates that the build-up of employee resources to better react to technostressors (i.e., secondary prevention) is often already feasible with only a few individuals. In contrast, measures initiating large-scale technological and organizational changes require more advanced organizational structures and larger organizations.

Lastly, the characteristics of *implementation duration*, *time from implementation until effect realization*, and *effect duration* take a time perspective. The results suggest that half of the measures can be implemented and brought to operational use in less than one year (e.g., measure 16, *train mentors for digital topics*). In contrast, eleven measures require 1–3 years, and one measure even three years (measure 11, *Develop team norms for the use of ICTs*). These numbers indicate that the initial effort for many measures is seen as relatively low, which is important for reducing the barrier to successful prevention. Similarly, the time required until the effect of the respective measure becomes apparent in operational business is estimated as less than half a year for ten measures (e.g., measure 21, *train technostress coping competencies*), up to one year for 13 measures, and more than one year for only one measure (measure 6, *foster a cooperative culture*). The effect of six technostress prevention measures is characterized to last less than one year, while 18 measures show a positive effect between one to three years (e.g., measure 1, *focus the ICT landscape*). Regarding primary vs. secondary technostress prevention measures, a clear tendency can be observed. The *average implementation duration* and *time until effect realization* are longer for primary than secondary technostress prevention. However, the *effect duration* for primary prevention was assessed to last 1–3 years for 14 of the 17 measures (~ 82%), while of the secondary technostress prevention measures, only four of seven measures (~ 57%) last 1–3 years and the rest less than one year.

Next, we asked the experts for a relevance assessment on which primary technostress prevention measures are expected to target which technostressor. For each technostressor, we identify two to six measures that the experts assessed as the most relevant. The resulting scores serve as a basis for confirmative quantitative statistical analyses on the relevance of different technostress prevention measures on the set of technostressors. Table 4 presents these relations and graphically displays the average relevance (scale from 0 to 6) of the measures for the respective technostressor for all primary technostress prevention measures. The measures are sorted according to their accumulated relevance score. Table 10 in the appendix presents the complete list of numerical relevance ratings and the number of experts who assessed the measure as highly relevant.

**Table 4:**
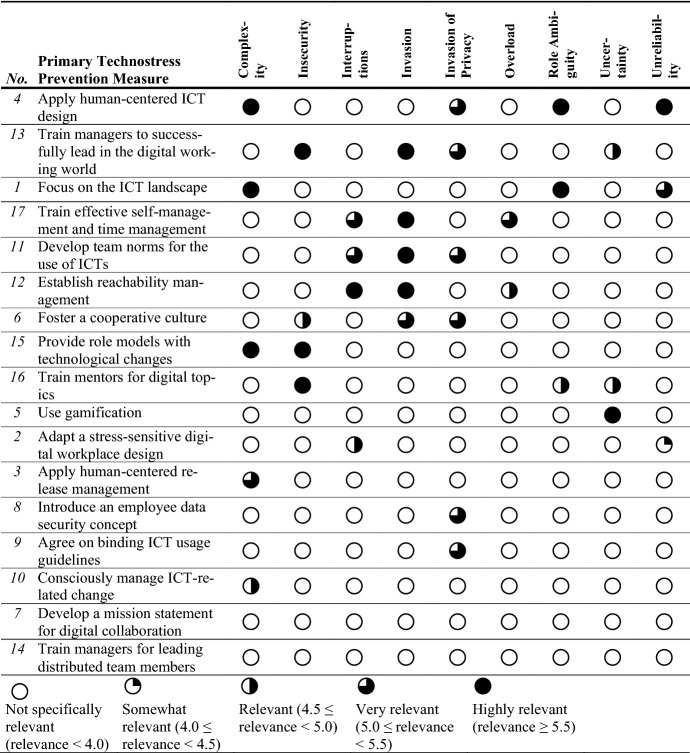
Relevance of primary prevention measures to reduce technostressors

When assessing the relations, no two technostressors share the same set of relevant technostress prevention measures. However, some patterns exist. For example, two technostress prevention measures can potentially help prevent complexity, unreliability, and role ambiguity. Both prevention measures (numbers 4 and 1) target the technological environment. When they reduce the complexity and unreliability of the technology, the need for employees to acquire new skills for working with complex and unreliable technology (and, hence, role ambiguity) are reduced. Similarly, interruptions and invasions share multiple prevention measures. Accordingly, these pairs of technostressors can be addressed through similar prevention measures.

Each technostress prevention measure is selected as a technostressor’s most relevant measure between zero to four times. Interestingly, two primary technostress prevention measures (measure 14, *train managers for leading distributed team members*, and measure 7*, develop a mission statement for digital collaboration*) are not selected to be among the most relevant for any technostressor. This result does not mean that the measures are generally not relevant for preventing technostress. However, it does indicate that industry experts do not expect them to be among the most relevant measures for addressing one of the nine considered technostressors. In contrast, two technostress prevention measures (measure 4, *apply human-centered ICT design*, and measure 13, *train managers to successfully lead in the digital working world*) are assessed as very or highly relevant to four different technostressors. This finding indicates that the two are rather general technostress prevention measures that are suitable for multiple technostressors. In that, measure 4 relates to changing the technological environmental conditions and measure 13 relates to the prominent role of leadership in preventive stress management (i.e., principle 2 of (techno)stress management). Other measures (e.g., measure 9, *agree on binding ICT usage guidelines*, and measure 10, *consciously manage ICT-related change*) are only relevant to one technostressor. These measures are expected to be specialized in addressing a particular technostressor.

## Propositions on preventive technostress management

Based on our results, we provide five propositions on preventive technostress management. The propositions are an abstraction of the results presented above. We see propositions as proposed relationships between constructs on the theoretical plane as compared to hypotheses on relationships between variables on the empirical plane (Bhattacherjee 2012, Chapter 2). The propositions are theoretical in the sense of relating to the understanding of a theory as relationships among constructs along with arguments substantiating the relationships and a definition of the scope of validity of the relationships (Whetten 1989). The purpose of the propositions here is to provide statements on potential relationships between technostress prevention constructs that stem from our empirical results and are compatible with existing theory on technostress and preventive stress management. The propositions are tentative and conjectural relationships between constructs; they need further theorizing and, after being transferred to research hypotheses, empirical testing for being fully integrated in theories of technostress. The propositions relate to abstract constructs on the theoretical plane. Hence, they cannot be tested empirically directly. Nevertheless, the propositions can serve as a foundation for developing testable hypotheses in the form of relationships between variables on the empirical plane (Bhattacherjee 2012, Chapter 2). The empirical test of hypotheses derived from the propositions is an indirect test of the propositions that will allow to reflect on their truth and usefulness. Once validated through the corresponding empirical test of related hypotheses, the propositions may serve as basis for designing preventive technostress management tools, methods, and programs.

For all following propositions, the scope is technostress in a work context with the organization as the entity that can prevent technostress among its employees. The arguments substantiating the proposed relationships derive from the contextualization of the Theory of Preventive Stress Management to technostress (Sect. 3) and the results of the literature analysis and the Delphi study (Sect. 5).

### Proposition 1

*The five principles of preventive stress management also apply to technostress.*

Quick and Quick (1979) intensively discuss work stress and its prevention. Given their publication before the widespread dissemination of digital technologies to work places, technology is not explicitly in focus. Quick et al. (1997) however do shortly discuss the role of early production technologies and a mismatch between people, task, technology, and structure as factors to consider in work stress research. Therein, the principles of preventive stress management are expected to apply to technostress, as technostress is a specific form of general stress. As such, the proposition is straightforward and in line with existing theory. Nevertheless, we suggest that stating it explicitly is important to direct the attention of technostress researchers towards (principle 1) the interdependence of individual and organizational health, (principle 2) the responsibility of leaders for individual and organizational health, (principle 3) the ability to prevent (or inhibit) individual and organizational stress, and (principle 5) the need for constant adaptation of preventive technostress management to keep up with ever-changing and dynamic organizations (Quick et al. 1997). Additional empirical evidence for the relevance of principles 2 and 3 can be found in the presence of prevention measures solely relevant to leadership (measures 13–15, principle 2), as well as the overall positive Delphi expert assessment of many prevention measures regarding their effectiveness in preventing technostress (principle 3). Principle 4 refers to the individuality of organizations and persons in their reaction to technostress (Quick et al. 1997). We do not see a need to direct attention to this principle as it is already front and center in technostress research (Beaudry and Pinsonneault 2005; Chen et al. 2019; Pirkkalainen et al. 2019; e.g., Salo et al. 2020).

### Proposition 2

*Technostress prevention measures address the technology, the organization, and/or the individual. Secondary technostress prevention measures focus primarily on the individuals.*

The differentiation of addressing technology, organizations, and/or individuals in prevention derives from literature (Murphy and Sauter 2004; Quick et al. 1997). Historically, the focus was mainly on distinguishing individuals and organizations (Quick et al. 1997). However, with the digitalization of work, the specific attention to technologies as a central component of work stress has emerged (e.g., Ayyagari et al. 2011; Ragu-Nathan et al. 2008; Tarafdar et al. 2019). This general differentiation is empirically supported by the developed set of technostress prevention measures in this study, given that it spans all three categories. The focus of secondary prevention on individuals is primarily induced from the characterization of the technostress prevention measures in this study. Five of the seven (71%) secondary prevention measures concern the individual level. Further, this insight integrates well with existing stress prevention theory. Secondary technostress prevention targets individuals’ technostress responses, i.e., physiological, psychological, and behavioral responses of individuals experiencing technostress (Quick et al. 1997). The behavioral response might be influenced by the technology or organization. However, physiological and psychological response are happening within the individual – hence the focus of respective prevention measures on individuals (LaMontagne et al. 2007; Salo et al. 2017). Omissions in proposition 2 are also noteworthy: The proposition does not refer to addressing the environment, customers, the employees’ family or the like. Thus, the proposition suggests a clear focus for identifying and designing further technostress prevention measures.

### Proposition 3

*Primary prevention measures differ with respect to the number of technostressors they are relevant for.*

Focusing on the relationship between technostressors and primary technostress prevention measures, we found patterns regarding the relevance of primary technostress prevention measures to reduce the frequency, duration, or/and intensity of technostressors. Our results indicate that not every measure addresses all technostressors equally. Some measures are relevant for one or a few specific technostressors, while other measures target a broader set of technostressors. For example, measure 8 (Introduce an employee data security concept) is explicitly concerned with increasing data security and reducing related risks. The measure is highly focused on one specific downside of digitalized workplaces and, as a result, targets only one technostressor: Invasion of privacy. In contrast, measure 13 (Train managers to successfully lead in the digital working world) addresses the very general topic of digital leadership. Strong digital leaders can help improve technostress stemming from all types of technostressors by identifying sources of technostress in time. The measure thus affects several technostressors (i.e., insecurity, invasion, invasion of privacy, and uncertainty). Table 4 gives the first indication of this matching based on the Delphi study’s expert assessment, which requires future empirical research to verify. While existing research has not yet systematically matched the full list of technostressors with the list of technostress prevention measures, few studies have investigated the effectiveness of some subset of technostress prevention measures for certain technostressors (e.g. Galluch et al. 2015; Schmidt et al. 2021; Valta et al. 2021). Their work supports the proposition that technostress prevention measures support different numbers of technostressors. For example, Valta et al. (2021) demonstrate social support systems and contact persons as measures addressing many technostressors. In contrast, the reduction of e-mail traffic is shown to be very useful only for two of their technostressors. In conclusion, we see a need for future research to go beyond our Delphi study’s relevance assessment and empirically establish the effectiveness of the different measures. Supporting technostress prevention in practice will require developing a body of knowledge on how to best select and implement a portfolio of technostress prevention measures which addresses the most important technostressors in a given organization.

### Proposition 4

*Technostressors differ with respect to whether they are addressable through only a few or many primary technostress prevention measures.*

Considering technostressors as the baseline (columns in Table 4), a few observations emerge from the empirical results in our study. Not all technostressors share the same number and relevance levels of technostress prevention measures through which they can be targeted. Consequently, some technostressors might be more difficult to address, requiring highly specialized measures for prevention. Others, however, exhibit a larger set of measures as potential prevention tools. For example, invasion is indicated to be addressable through several different measures. The technostressor addresses a vague feeling of having to be connected and there are many ways to change this perception. Unreliability, on the other hand, is only matched with three measures in our study. As unreliability describes technostress from technical failure, the options for prevention are more limited: improve technical reliability or improve coping with failures. Complementing our observations, Valta et al. (2021) has highlighted results that support this hypotheses. In their study, for example, overload is demonstrated to be addressable by multiple technostress prevention measures in their sample, while cyberbullying is only addressed by one. As with proposition 3, we deem it important to point to this multifaceted interplay of primary technostress prevention measures and technostressors to spur future research in the direction of providing knowledge on managing a portfolio of technostress prevention measures.

### Proposition 5

*Compared to secondary prevention, primary technostress prevention measures represent a longer-term approach with a longer effect duration, but also require higher initial efforts.*

Deep-diving into the characteristics of primary and secondary prevention measures in Table 8 of the appendix, we identified that primary measures tend to require higher average initial efforts for implementation and effect realization than secondary prevention measures. While for primary prevention only six out of seventeen (35%) technostress prevention measures require less than one year for implementation, six out of seven (86%) secondary technostress prevention measures were characterized with the shortest implementation time category. Simultaneously, however, the experts in our study indicated that primary technostress prevention measures yield longer-term positive effects. This leads to the proposition that primary technostress prevention is more focused on a long-term approach to preventing technostress, while secondary technostress prevention measures on average present a short- and mid-term solution to preventing technostress. Existing literature has not yet analyzed the differences between effect and implementation duration when comparing primary and secondary prevention. However, the nature of both prevention types offers some arguments supporting this proposition. Secondary prevention measures mostly address individuals and their response to technostressors, including their skills, competencies and experiences (Salo et al. 2017). Trainings and workshops that improve an individual’s coping or IT skills can be conducted within a matter of hours or days thus leading to a low implementation and effect duration effort. On the downside, high employee turnover rates, particularly in IT professionals, lead to a frequent loss of knowledge and skills gained from secondary technostress prevention measures and reduce the effect duration period in this prevention type (Wang et al. 2022). Primary prevention measures on the other side often target the technological and organizational environment (Quick et al. 1997; Salo et al. 2017). Therein, changes stemming from prevention measures often affect overarching organizational and technological structures comprising many people and entities. While this makes their implementation more complex and time-consuming, it also reduces the dependency on individuals, thus increasing the effect duration period.

## Discussion

Reducing the adverse outcomes of technostress is essential. In an ideal world, organizations invest in addressing technostress at an early level and throughout the process (Brivio et al. 2018). To sustainably reduce technostress, organizations need to implement ex-ante measures to prevent future stressful situations caused by technostressors, and they need to support their employees in responding to technostress. To drive knowledge on prevention in the technostress field and assist practitioners in implementing successful technostress prevention management, we bring together different research strands of technostress inhibition and stress management and enrich the combined perspective empirically. With our study, we follow Tarafdar et al.'s (2019) call for 1) a more thorough investigation of how altering technological aspects in an organizational environment can prevent technostress and 2) applying a methodological approach in technostress research that complements the current focus of technostress research on surveys.

### Contributions and implications for research

The study contributes to research in four ways:

1. Embedding technostress inhibitors in the larger context of preventive technostress management
2. Providing a structured overview of 24 technostress prevention measures
3. Characterizing of the 24 technostress prevention measures in terms of their basic approach to preventive technostress management, their applicability in practice, and their relevance in targeting technostressors.
4. Formulating propositions on preventive technostress management

First, we embed existing knowledge on technostress inhibition in the larger context of preventive technostress management by bringing together theory on technostress inhibitors (e.g., Ragu-Nathan et al. 2008; Sarabadani 2018) with the three types of stress prevention (primary, secondary, tertiary) from the Theory of Preventive Stress Management. The research strand of technostress inhibition theorizes inhibitors to either (1) act as antecedents to technostressors (Jena 2015; Tarafdar et al. 2011, 2015), moderate the relationship between technostressor and outcomes (Ragu-Nathan et al. 2008), or decrease adverse outcomes directly (Jena 2015; Ragu-Nathan et al. 2008; Tarafdar et al. 2011). We synthesize this existing knowledge and combine it with insights of the Theory of Preventive Stress Management which are very similarly located in the general stress process as the three theorizations of technostress inhibition. For example, Tarafdar et al. (2015) integrates technostress inhibitors as antecedents to technostressors, including the three inhibitors: “Facilitate technical literacy”, “provide technical support”, and “facilitate technology involvement”. While Ragu-Nathan et al. (2008) and Fuglseth and Sørebø (2014) mention the same three technostress inhibitors, the integration into the technostress process differs. Based on our definition of primary and secondary technostress dimension, we use these insights to summarize and structure applicable technostress prevention measures on the same level of detail in their role of the technostress process. Based on the insights of the different research strands of technostress inhibition and preventive stress management, we develop a conceptual model of preventive technostress management detailing the target points of the three types of technostress prevention. Hereby, we provide five guiding principles of technostress management. These are derived from the Theory of Preventive Stress Management and are new to the technostress literature.

Second, we provide a structured list of 24 actionable technostress prevention measures which consists of a synthesis of inhibitor literature (twelve technostress prevention measures), a literature-based expansion of eight further technostress prevention measures mentioned in related technostress literature, and an empirical expansion of four new technostress prevention measures. Therefore, while 20 of the 24 technostress prevention measures presented in this paper have in some form been mentioned in technostress literature (e.g., *provide ICT training* or *provide supportive ICTs*) (e.g. Adam et al. 2017; Pfaffinger et al. 2020), their domain varies between technostress inhibition (e.g., Fuglseth and Sørebø 2014; Tarafdar et al. 2015), mitigation (e.g., Day et al. 2012; Hung et al. 2015), coping (D’Arcy et al. 2014; e.g., Pirkkalainen et al. 2019), or other related constructs (e.g., Benlian 2020; Richter and Richter 2020). Regarding the research strand of technostress inhibition, the inhibitors named by, among others, Tarafdar et al. (2015), Ragu-Nathan et al. (2008), or Fuglseth and Sørebø (2014) were used as inputs for, among others, the prevention measures 3 *apply human-centered release management* (primary prevention), measure 19 *provide ICT support* (secondary prevention), and measure 23 *provide ICT training* (secondary prevention). Beyond the named technostress inhibitors, the articles mention further prevention measure candidates, that went into the description of our prevention measures. For example, next to the three above mentioned technostress inhibitors, Fuglseth and Sørebø (2014) state: “However, the study also indicates that managers should actively encourage employees to try out new ICT, and reward employees for using new ICT” and “In addition, managers should emphasize teamwork and encourage ICT knowledge sharing”. These insights went, among others, into the prevention measures 13 *train managers to successfully lead in the digital working world*, measure 15 *provide role models with technological changes*, and measure 22 *offer platforms to exchange experience on ICT use*. This example illustrates that our study goes beyond existing literature on technostress inhibitors and related studies. We expand on prior literature by intensively analyzing existing studies and collecting all possible content as prevention measure candidates to then synthesize them by clustering them on the same level of detail and discussing them with experts. The engagement with the experts results in adding further technostress prevention measures beyond literature. See Table 5 and Table 6 in the appendix for a complete overview of all references and their measures that served as an input for our technostress prevention measures.

The first two contributions lead to the following implications for research: Researchers working on analyzing or designing technostress inhibitors can use the prevention framing and approach literature on general stress prevention to obtain further theoretical grounding for their technostress research. Further, they can use our set and structure of technostress prevention measures as a broad overview of measures in the otherwise fragmented literature. Finally, researchers working on stress prevention in other domains besides technostress might consider our set of technostress prevention measures as inspiration for identifying similar prevention measures in other specific stress contexts or in abstracting them from technostress to general stress prevention research.

Moving to the third contribution, we structure and characterize all 24 measures in terms of their basic approach to technostress prevention (i.e., primary and secondary technostress prevention as well as the entity affected: technology, organization, or individual), their applicability, and their relevance in targeting technostressors (for primary measures). Thereby, we contribute a common starting ground for addressing technostress prevention from an organizational view. Due to their importance for future technostress research, we point out two specific facets of the set of characterized technostress prevention measures: The importance of leaders and the lack of a one-fits-all approach. The importance of leaders has been emphasized for preventive stress management (Macik-Frey et al. 2007; Quick 1992). Because of the critical role of leaders, it can be beneficial for organizations to also target primary stress prevention measures to leaders (e.g., executive coaching and peer support) (Hargrove et al. 2011). Arguably, leaders play a similarly important role in technostress prevention as in the prevention of stress in general. Looking at the most relevant primary technostress prevention measures, we find that measure 13, *Train managers to successfully lead in the digital working world* is the second most relevant (Table 4). Further concerning prior findings on primary stress prevention measures in general, the provision of instrumental (e.g., buddies or mentoring programs), informational (e.g., improvement of the flow of information), and emotional (e.g., increasing emotional understanding among employees) support proofed effective as primary preventive interventions (Quick and Quick 1984a). In the specific context of technostress, we also identified all types of the mentioned support measures with a specific focus on ICTs: Measure 15, *provide role models with technological changes,* and measure 16, *train mentors for digital topics* (8^th^ and 9^th^ most relevant primary technostress prevention measure), relate to the first category of “instrumental support”. Informational support is addressed by the third relevant technostress prevention measure, measure 1, *focus on the ICT landscape* (Table 4) by reducing the complexity and avoiding redundancies of information. Lastly, emotional support is mainly addressed by measure 6, *foster a cooperative culture* (6^th^ most relevant primary technostress prevention measure). While the most relevant primary preventive stress measures can be found in the nine most relevant primary technostress prevention measures, we additionally found purely ICT-specific measures like the most relevant measure 4, *Apply human-centered ICT design*.

Our efforts in characterizing the different technostress prevention measures in terms of their applicability also help structure the field of preventive technostress management. We offer the possibility to describe measures or groups of measures on a shared set of characteristics. This possibility is important to better compare and classify similar measures in future research. Technostress research can make use of this grouping by not having to assess every measure individually but by being able to assess groups of measures that are similar in their characteristics. We found that primary technostress prevention measures require high initial efforts but yield long-term effects. Therein, primary technostress prevention measures deem a suitable long-term approach for designing workplaces that are technostress free. In contrast, most secondary prevention measures target individuals and require fewer implementation efforts. Thus, they present an opportunity for short- and mid-term prevention of technostress by enabling employees to better react to technostressors in the phase before they have been eliminated permanently. For future research, the implementation and effect duration classification also help design more appropriate studies on testing the actual effectiveness of the different measures. For example, a measure that takes one year to create a positive effect cannot reasonably be assessed in a six-month field study.

Additionally, we provide initial evidence that technostress prevention measures are expected to address different technostressors, and prevention is no one-fits-all mechanism. Hereby, we related the primary technostress prevention measures to nine established technostressors to increase practical applicability and to build the foundation for extensive empirical analyses in future research. Therein, we built on, validated, and extended the few studies that assessed selected prevention measures' potential for specific technostressors (e.g. Galluch et al. 2015; Schmidt et al. 2021; Valta et al. 2021). For example, our results confirm and expand the findings of Valta et al. (2021), who investigated seven measures (e.g., ICT training, contact person) to reduce single technostressors. The authors found that, for example, homogenizing the ICT landscape (here: measure 1, *focusing the ICT landscape*) reduces the technostressor *complexity*. This goes along with our results (e.g., measure 1 is highly relevant for complexity, see Table 4). We expanded the results by finding more technostress prevention measures that are expected to be relevant for preventing complexity (e.g., measure 3 *apply human-centered release management*, measure 4 *apply human-centered ICT design*, and measure 10 *consciously manage ICT-related change*). Stemming from the descriptive results of the expert panel, our results serve as the first foundation for relevance. Scholars in the field can build on these results and further theorize on the relationship between individual technostress prevention measures (or sets of such measures with similar characteristics) on specific technostressors.

Finally, we contribute by abstracting our theoretical and empirical results to five propositions on preventive technostress management. These are contributions to technostress research, not to the general Theory of Preventive Stress Management. Combining our results with other such contextualizations of specific forms of stress, it might eventually become possible to consolidate the specific findings into a more general context-contingent theory of preventive stress management (Hong et al. 2014). Our five propositions are grounded in theorizing and empirical evidence. We call for future research engaging with these propositions to refute, refine, or validate them. The guiding principles of preventive technostress management and proposition 1 provide a new framing and a new lens to researching technostress at work. Future technostress research should further build on stress prevention theory to advance our knowledge on organizational health, the responsibility of leaders for health, and the constant management and adaptation of a portfolio of technostress prevention measures.

### Implications for practice

Organizations have a moral, legal, and economic obligation to address work-related technostress among employees. Moral in the sense of having the responsibility to offer a workplace that is safe and healthy. Legal in the sense that countries like Germany have established laws on an organization’s duty to protect employees' physical and psychological well-being. Economic in the sense that technostress can impair an individual’s health and work performance and negatively impact an organization’s performance (Tarafdar et al. 2015). As adverse outcomes from technostress are not acute events but develop over time, it is important to prevent technostress throughout the process, rather than only reacting after adverse outcomes arise. This indicates a need to support organizations in their prevention efforts by providing actionable fine-grained knowledge on preventive technostress management and measures to implement.

Our research demonstrates the diversity of available prevention measures and, thus, supports organizations in finding a set of measures applicable to their specific setting. Especially for organizations new to technostress prevention, our set of measures offers an information source to understand what different aspects prevention can comprise. Most measures are primary technostress prevention measures. This is noteworthy because secondary prevention is prevalent in job-related stress prevention practice, even though primary prevention is deemed more effective as it tackles stress at its source (LaMontagne et al. 2007). By offering a rich set of primary technostress prevention measures, we support organizations in establishing a comprehensive approach to preventive technostress management.

In terms of characterization, our study shows that most measures are available for organizations of all sizes. However, the measures differ strongly in the technostressors they address, their implementation costs, or their required expert knowledge. Further, most measures require relatively little time until implementation and effect realization. These are encouraging news for practice that might take away some of the burdens when starting with technostress prevention. The characterization of measures also serves as decision support for organizations. When choosing measures, one can easily filter measures by the relevant criteria (e.g., size or entity of change) to be presented with a set of appropriate measures.

The results on the difference in relevance of single technostress prevention measures and specific technostressors have substantial implications for practice. The relevance of technostress prevention measures is expected to be significantly impacted by the technostressor responsible for the technostress. Hence, as the first step to technostress prevention, organizations must identify the critical technostressors within their workforce. Only once an organization knows which technostressors cause technostress to what extent they can effectively select prevention measures. Different measures or a portfolio of prevention measures might be appropriate depending on whether one or multiple technostressors are prevalent. Especially if resources are scarce (typically), it is crucial to prioritize the most severe technostressors concerning prevention.

### Limitations

Like any research paper, our study is subject to limitations. First, our results are based on the expertise of a limited number of 13 panelists and, before the Delphi study, a structured literature review and 17 experts in focus groups. We are convinced that our panel is diverse because panelists stem from different organizations of different industries. Still, we can make no formal claim about the panel’s representativeness. Even though our Delphi study panel’s structure and size fit our research purpose, the measures’ relevance (Table 4) should be interpreted only as a rough first estimate. These relevance statements need to be evaluated in more extensive quantitative empirical research in the future to make definitive, generalizable claims. The effectiveness of a technostress prevention measure may highly depend on individual and organizational differences and characteristics (principle 5). For example, demographics, professional experience, or computer self-efficacy potentially affect how strongly people experience technostress and how effective a prevention measure is. To address this, future research should also assess the significance of such confounding variables, similar to existing studies such as Tams et al.'s (2018) work.

Second, we examined the assumed effects of primary technostress prevention measures on nine technostressors. While the selection comprises the most frequently studied technostressors, the literature holds further technostressors (Maier et al. 2012). Also, since the research field is currently rapidly advancing, new technostressors were proposed in the meantime. One example is Fischer et al. (2021), who developed ten technostressor categories, including new constructs like social environment or technical support. Consequently, some newer technostressors found no consideration in the Delphi study. Further technostressors could be included in future works.

Third, we did not narrow the organizational scope to industries or company sizes. We kept the scope broad because this overview of technostress prevention is aimed to be relevant for all types of organizations. However, some of our results might depend on the respective industry, especially concerning primary technostress prevention measures. This should be considered when applying the measures in organizations, and an additional individualization of measures to industries could be required.

Fourth, our study derives work-related technostress prevention measures that enable technological, organizational, and individual changes. On the technological level, the prevention measures are mostly generic in that they generally refer to ICTs. It would be valuable to assess specific ICTs (e.g., e-mail applications or data management software) as a next step. One could also develop additional prevention measures that target any technostress from the respective ICTs or group of ICTs.

Fifth, in this paper, we address individual technostress prevention measures. In real-life scenarios, organizations will apply portfolios of such measures. To increase applicability, it would thus be precious to create a) quantification of the effect of different prevention portfolios (including any positive and negative interdependencies) and b) a handbook/ guideline on how to develop and implement such a portfolio in each setting.

Finally, we derive propositions of preventive technostress management, which have an empirical basis in our study. However, they should be transformed to research hypotheses for empirical testing in future research.

## Conclusion

Understanding technostress and its adverse outcomes have emerged as a popular and important research endeavor. Research has extensively investigated the potential effects of technostress on employees’ health and organizational performance (Fuglseth and Sørebø 2014; Ragu-Nathan et al. 2008; Srivastava et al. 2015; Tarafdar et al. 2007, 2015). Further valuable studies have also looked into ways of inhibiting the adverse outcomes of technostress (e.g. Ragu-Nathan et al. 2008; Weinert et al. 2020).

Our study complements existing research in that we combine the research strands of technostress inhibition and further related technostress-reducing literature with preventive stress management. Thereby, we focus on addressing the ex-ante prevention of technostress from an organizational perspective. We apply a Delphi study yielding a set of 24 relevant prevention measures that specifically address work-related technostress. Our study characterizes these measures concerning their basic approach to preventive technostress management, applicability in practice (e.g., organizational size, target group, duration of implementation, realization duration, and effect duration) and their relevance in targeting technostressors. On a more abstract level, we provide guiding principles and proposition for preventive technostress management. This paper's contribution lies in embedding technostress inhibitors in the larger context of preventive technostress management by creating a theoretical basis for technostress prevention, a unification of existing prevention and technostress mitigation or inhibition studies, and a step towards structuring the dynamics underlying prevention measures. For organizations, we offer valuable support to fulfill their moral, legal, and economic responsibility to reduce technostress among their employees.

## Data Availability

The data that support the findings of this study are available from the corresponding authors upon reasonable request.

## Appendix

See Figs. 5 and Tables 5, 6, 7, 8, 9 and 10.

**Table 5:** Result of literature review (technostress inhibitor article)

Study	Context	Mentioned potential prevention measures	Used as an input for following prevention measure
Bala and Venkatesh (2016)	Work-related technostress	Experiential engagements (user participation, training effectiveness) and psychological engagements (user involvement, management support)	(3) Apply human-centered release management (4) Apply human-centered ICT design (13) Train managers to successfully lead in the digital working world (15) Provide role models with technological changes (23) Provide ICT training
Chandra et al. (2019)	Technostress and employee innovation	Sensibilization, training, social support (mentor)	(16) Train mentors for digital topics (23) Provide ICT training (24) Foster sensitization and self-reflection regarding technostress
Fuglseth and Sørebø (2014)	Work-related technostress	Helpdesk, supportive leadership style, end-user training, user involvement, knowledge sharing	(3) Apply human-centered release management (19) Provide ICT support (11) Develop team norms for the use of ICTs (23) Provide ICT training (13) Train managers to successfully lead in the digital working world (15) Provide role models with technological changes (22) Offer platforms to exchange experience on ICT use
Ragu-Nathan et al. (2008)	Work-related technostress	Technical support provision, literacy facilitation, involvement facilitation	(3) Apply human-centered release management (4) Apply human-centered ICT design (6) Foster a cooperative culture (19) Provide ICT support (10) Consciously manage ICT-related change (23) Provide ICT training (15) Provide role models with technological changes
Tarafdar et al. (2010)	Work-related technostress	User involvement in the design of IS, support mechanisms for innovation	(3) Apply human-centered release management (4) Apply human-centered ICT design
Tarafdar et al. (2011)	Work-related technostress	Technical support provision, technology involvement facilitation, innovation support	(3) Apply human-centered release management (4) Apply human-centered ICT design (19) Provide ICT support
Tarafdar et al. (2015)	Work-related technostress	Self-efficacy, technical support (helpdesk), technology competence, user involvement	(3) Apply human-centered release management (19) Provide ICT support (23) Provide ICT training

**Table 6:** Result of the literature review (further related technostress articles)

Study	Context	Mentioned potential prevention measures	Used as an input for following prevention measure
Adam et al. (2017)	Work-related technostress	Stress-sensitive adaptive enterprise systems	(18) Provide supportive ICTs
Al-Fudail and Mellar (2008)	Technostress of teachers	Resources (reliable technology), social support (mentoring), technical support, IT training	(16) Train mentors for digital topics (19) Provide ICT support (23) Provide ICT training
Arnetz (1996)	Work-related technostress	Healthy and productive design of the workplace, training programs	(2) Adapt a stress-sensitive digital workplace design (23) Provide ICT training
Benlian (2020)	Technostress spillovers from work to home	Social support (coaching), facilitate employees' awareness of technostress through mentoring, supportive culture concerning accessibility, management leadership	(6) Foster a cooperative culture (7) Develop a mission statement for digital collaboration (13) Train managers to successfully lead in the digital working world (16) Train mentors for digital topics (24) Foster sensitization and self-reflection regarding technostress
Cao and Yu (2019)	Technostress through social media at work	Sensibilization and awareness to enable behavior changes for social media usage that is actively managed	(17) Train effective self-management and time management (24) Foster sensitization and self-reflection regarding technostress
Caro and Sethi (1985)	Work-related technostress	Technological planning, culture, technostress monitoring systems, self-development programs	(3) Apply human-centered release management (18) Provide supportive ICTs (6) Foster a cooperative culture (23) Provide ICT training
Carolan et al. (2017)	Work-related technostress	Discussion groups, time management, information on stress	(17) Train effective self-management and time management (22) Offer platforms to exchange experience on ICT use (24) Foster sensitization and self-reflection regarding technostress
Chen et al. (2019)	Technostress through mobile apps in leisure time	Sensibilization, self-reflection, time management, monotasking	(17) Train effective self-management and time management (20) Train monotasking (24) Foster sensitization and self-reflection regarding technostress
D’Arcy et al. (2014)	Security-related technostress	Helpdesk, awareness programs, education, and awareness security policies	(18) Provide supportive ICTs (6) Foster a cooperative culture (8) Introduce an employee data security concept (19) Provide ICT support (11) Develop team norms for the use of ICTs (23) Provide ICT training (24) Foster sensitization and self-reflection regarding technostress
Day et al. (2012)	Work-related technostress	Assistance like helpdesk, mentor, leader, culture, and clear communication guidelines	(18) Provide supportive ICTs (6) Foster a cooperative culture (7) Develop a mission statement for digital collaboration (19) Provide ICT support (13) Train managers to successfully lead in the digital working world (15) Provide role models with technological changes (16) Train mentors for digital topics
Delpechitre et al. (2019)	Salespeople's technostress	Increase self-efficacy	(23) Provide ICT training
Elie-Dit-Cosaque et al. (2011)	Work-related technostress	Top management support, supportive leadership style	(13) Train managers to successfully lead in the digital working world (15) Provide role models with technological changes
Galluch et al. (2015)	Work-related technostress	Timing control, method control, resource control	(11) Develop team norms for the use of ICTs (17) Train effective self-management and time management
Hung et al. (2015)	Personality and technostress	Proactive personality traits (training, self-reflection)	(24) Foster sensitization & self-reflection regarding technostress
Khedhaouria and Cucchi (2019)	Technostress of senior managers	Self-management, time-management, availability, supportive leadership style	(12) Establish reachability management (17) Train effective self-management and time-management (13) Train managers to successfully lead in the digital working world (20) Train monotasking
Kim and Kim (2014)	Work-related technostress	Supportive leadership style, helpdesk	(19) Provide ICT support (13) Train managers to successfully lead in the digital working world
Kloker (2020)	Technostress in daily lives	Situation management, gratification management, expectation management	(24) Foster sensitization and self-reflection regarding technostress
Lee et al. (2015)	Technostress in general	Interaction with indoor plants	(2) Adapt a stress-sensitive digital workplace design
Manning et al. (2020)	Technostress of high school teacher	Trustful culture, leading style	(18) Provide supportive ICTs (6) Foster a cooperative culture (13) Train managers to successfully lead in the digital working world
Pfaffinger et al. (2020)	Work-related technostress	Expectation management, training, self-management, time-management, leadership style, helpdesk	(11) Develop team norms for the use of ICTs (19) Provide ICT support (12) Establish reachability management (23) Provide ICT training (17) Train effective self-management and time management (13) Train managers to successfully lead in the digital working world (14) Train managers for leading of distributed team members
Pirkkalainen et al. (2019)	Work-related technostress	Training to raise awareness, encourage self-reflection, and self-management	(4) Apply human-centered ICT design (23) Provide ICT training (24) Foster sensitization and self-reflection regarding technostress (17) Train effective self-management and time management
Revilla Munoz et al. (2016)	Technostress of Teachers	Support program	(19) Provide ICT support (22) Offer platforms to exchange experience on ICT use
Richter and Richter (2020)	Technostress of digital nomads	Expectation management	(7) Develop a mission statement for digital collaboration (11) Develop team norms for the use of ICTs
Saunders et al. (2017)	Techno-overload	Monotasking	(20) Train monotasking
Saxena and Lamest (2018)	Technostress on a business manager	Personal and organizational coping (supportive systems, monotasking, training, helpdesk)	(18) Provide supportive ICTs (7) Develop a mission statement for digital collaboration (19) Provide ICT support (17) Train effective self-management and time management (13) Train managers to successfully lead in the digital working world
Stich et al. (2019)	Technostress through E-mail Usage	Self-reflection on e-mail usage, expectation management concerning individual's preferences concerning e-mail overload	(7) Develop a mission statement for digital collaboration (11) Develop team norms for the use of ICTs (24) Foster sensitization and self-reflection regarding technostress
Tarafdar et al. (2007)	Work-related technostress	ICT training, communication about the need of ICT, leadership style, discussion forums	(23) Provide ICT training (13) Train managers to successfully lead in the digital working world (15) Provide role models with technological changes (22) Offer platforms to exchange experience on ICT use
Turel and Gaudioso (2018)	Work-related technostress	Supportive leadership style and culture with less competition	(6) Foster a cooperative culture (11) Develop team norms for the use of ICTs (13) Train managers to successfully lead in the digital working world
Weinert et al. (2020)	Individual perspective of technostress mitigation	Instrumental and social support	(19) Provide ICT support (16) Train mentors for digital topics
Weinstein et al. (2016)	Technostress in daily lives	Online discussion forums	(22) Offer platforms to exchange experience on ICT use
Wiholm et al. (2000)	Work-related technostress	Stress management training	(24) Foster sensitization and self-reflection regarding technostress (17) Train effective self-management and time management
Yu et al. (2017)	Technology adaption	Training, user involvement for selection of media, and the design of technology	(17) Train effective self-management and time management (23) Provide ICT training
Yun et al. (2012)	Office-home smartphones and technostress	Supportive culture to minimize work-to-life conflict and tools to support this	(6) Foster a cooperative culture (18) Provide supportive ICTs

**Table 7:** Participants of the focus group workshops

Code	Role	Workshop
Part1	Researcher focusing on the digitalization of the workplace working for a German university	First
Part2	Head of human resources department in an SME focusing on customer acquisition and retention	First
Part3	Member of the works council working for a manufacturer of entertainment and communication technology with over 2,000 employees	First
Part4	An employee working for an SME focusing on customer acquisition and retention	First
Part5	Professor for business and information systems engineering at a German university	First
Part6	A person in charge of psychological risk assessments working for an occupational health management service provider responsible for over one million employees	Both
Part7	Consultant with a focus in working-time and work organization focusing on SME	Both
Part8	Psychologist and trainer working for an occupational health management service provider responsible for over one million employees	Both
Part9	An emergency psychologist working for an occupational health management service provider responsible for over one million employees	Second
Part10	A researcher with a focus in working-time and work organization at a federal institute focusing on occupational safety and health	Second

**Table 8:** Delphi study panelists

Code	Role
Exp1	Senior ombudsperson for severely disabled persons at an aircraft manufacturer responsible for health management
Exp2	Experienced employer representative working for the employers' association for a major industry in the country
Exp3	Occupational physician in the field of healthiness and well-being for a large workers' compensation company
Exp4	Former vice-chairman of a works council and lecturer at a training institute for works councils focusing among others on remote work and stress management
Exp5	Employee representative working for a national trade union center for a large trade union in the country
Exp6	Scientific director of a federal institute focusing on occupational safety and health management
Exp7	Senior researcher at an institute focusing on remote work and qualification
Exp8	Head of health management at the human resources department for a company in the construction industry
Exp9	Senior safety specialist at the human resources department for a company in the construction industry responsible for corporate health measures, among others stress management and remote work
Exp10	Occupational safety specialist at the human resources department for a company in the baking industry, responsible for stress management in the company
Exp11	Systemic consultant in and with organizations for a company in the baking industry regarding health and stress management
Exp12	Head of occupational safety at the human resources department for a company in the construction industry responsible for corporate health measures, among others during remote work
Exp13	26 years experience with psychosocial counseling and psychological coaching for employees and managers in large companies as a social consultant with a focus on, e.g., work-life-balance, stress, remote working

**Table 9:** Characterization of prevention measures in terms of their basic approach to technostress prevention and their applicability in practice

	(I//II)			
#	Technostress prevention measure	Prevention type	Entity of change	Target group
1	Focus the ICT landscape	Primary	Technology	All
2	Adapt a stress-sensitive digital workplace design	Primary	Technology	All
3	Apply human-centered release management	Primary	Technology	All
4	Apply human-centered ICT design	Primary	Technology	All
5	Use gamification	Primary	Technology	All
6	Foster a cooperative culture	Primary	Organization	All
7	Develop a mission statement for digital collaboration	Primary	Organization	All
8	Introduce an employee data security concept	Primary	Organization	All
9	Agree on binding ICT usage guidelines	Primary	Organization	All
10	Consciously manage ICT-related change	Primary	Organization	All
11	Develop team norms for the use of ICTs	Primary	Organization	All
12	Establish reachability management	Primary	Organization	All
13	Train managers to successfully lead in the digital working world	Primary	Individual	Mgmt
14	Train managers for leading of distributed team members	Primary	Individual	Mgmt
15	Provide role models with technological changes	Primary	Individual	Mgmt
16	Train mentors for digital topics	Primary	Individual	All
17	Train effective self-management and time management	Primary	Individual	All
18	Provide supportive ICTs	Secondary	Technology	All
19	Provide ICT support	Secondary	Organization	All
20	Train monotasking	Secondary	Individual	All
21	Train technostress coping competencies	Secondary	Individual	All
22	Offer platforms to exchange experience on ICT use	Secondary	Individual	All
23	Provide ICT training	Secondary	Individual	All
24	Foster sensitization and self-reflection regarding technostress	Secondary	Individual	All

	(II/II)			
#	Organization size	Implementation duration	Realization duration	Effect duration
1	Min. 50	1–3 years	Less than 0.5 years	1–3 years
2	Min. 10	1–3 years	Less than 0.5 years	1–3 years
3	Min. 50	Less than 1 year	0.5–1 year	1–3 years
4	Min. 50	Less than 1 year	Less than 0.5 years	1–3 years
5	Min. 50	1–3 years	0.5–1 year	Less than 1 year
6	Min. 50	1–3 years	More than 1 year	1–3 years
7	Min. 50	1–3 years	0.5–1 year	1–3 years
8	Min. 10	Less than 1 year	Less than 0.5 years	1–3 years
9	Min. 50	1–3 years	0.5–1 year	1–3 years
10	Min. 50	1–3 years	0.5–1 year	1–3 years
11	Min. 50	More than 3 years	Less than 0.5 years	1–3 years
12	Min. 10	Less than 1 year	0.5–1 year	Less than 1 year
13	Min. 50	1–3 years	0.5–1 year	1–3 years
14	Min. 50	1–3 years	0.5–1 year	1–3 years
15	Min. 50	1–3 years	0.5–1 year	1–3 years
16	Min. 250	Less than 1 year	Less than 0.5 years	1–3 years
17	Min. 10	Less than 1 year	0.5–1 year	Less than 1 year
18	Min. 10	Less than 1 year	Less than 0.5 years	Less than 1 year
19	Min. 250	Less than 1 year	Less than 0.5 years	1–3 years
20	Min. 10	Less than 1 year	Less than 0.5 years	1–3 years
21	Min. 10	Less than 1 year	Less than 0.5 years	Less than 1 year
22	Min. 50	Less than 1 year	0.5–1 year	1–3 years
23	Min. 10	1–3 years	0.5–1 year	1–3 years
24	Min. 10	Less than 1 year	0.5–1 year	Less than 1 year

**Table 10:** Detailed results on the relevance of primary prevention measures to reduce technostressors

PM	Complexity	Insecurity	Interruptions	Invasion	Invasion of Privacy	Overload	Role Ambiguity	Uncertainty	Unreliability	∑ Stressors
1	6,00 (11x)						5,92 (13x)		5,08 (13x)	**3**
2			4,85 (13x)						4,00 (11x)	**2**
3	5,00 (10x)									**1**
4	6,00 (13x)				5,00 (11x)		5,92 (13x)		6,00 (13x)	**4**
5								5,58 (12x)		**1**
6		4,92 (13x)		5,00 (13x)	5,09 (11x)					**3**
7										**0**
8					5,23 (13x)					**1**
9					5,15 (13x)					**1**
10	4,92 (12x)									**1**
11			5,08 (13x)	5,83 (12x)	5,00 (12x)					**3**
12			6,00 (13x)	6,00 (13x)		4,83 (12x)				**3**
13		6,00 (12x)		5,85 (13x)	5,00 (11x)			4,92 (13x)		**4**
14										**0**
15	5,92 (13x)	5,92 (13x)								**2**
16		6,00 (12x)					5,08 (12x)	5,15 (13x)		**3**
17			5,00 (13x)	5,92 (12x)		5,17 (12x)				**3**
**∑**	**5**	**4**	**4**	**5**	**6**	**2**	**3**	**3**	**3**	

*PM* prevention measure

## Footnotes

1 German Occupational Safety and Health Act of 7th August 1996 (Federal Law Gazette p. 1246), as amended by Article 1 of the Act of 22 December 2020 (Federal Law Gazette p. 3334).

2 Riedl et al. (2012) focus on physiological responses, Califf et al. (2020) on psychological responses, Tarafdar et al. (2019) on coping responses. In Fig. 1, we follow Lazarus and Folkman (1984) in summarizing these types of responses as a general (techno)stress response, since these different responses are interdependet and not clearly separable in time.

3 The original naming used the prefix “techno-” “for each of these technostressors. We omit this prefix for brevity. Tarafdar et al. (2007), Ragu-Nathan et al. (2008) and others use the term ‘technostress creator’ rather than ‘technostressor.’ We treat these terms as synonyms and follow Tarafdar et al. (2019) in using technostressor.

## Code availability

Code for data cleaning and analysis are available from the corresponding authors upon reasonable request.
